# Triptolide in Combination with the Gli1 Inhibitor GANT61 Eradicates Anaplastic Thyroid Cancer by Suppressing Cancer Stem Cell Self-Renewal and Enhancing Apoptosis and Pyroptosis

**DOI:** 10.7150/ijbs.131070

**Published:** 2026-09-10

**Authors:** Yuqing Zhao, Yue Zhu, Shuying Zhong, Penggang Liu, Wei Li, Xiaoyun Mao, Xiulong Xu

**Affiliations:** 1 Institute of Comparative Medicine, College of Veterinary Medicine, Yangzhou University, Yangzhou 225009, China.; 2 State Key Laboratory of Molecular Oncology, Peking University Cancer Hospital and Institute, Peking University International Cancer Institute, Frontiers Science Center for Cancer Integrative Omics, Peking University Health Science Center, Beijing 100191, China.; 3 School of Basic Medical Sciences & School of Public Health, Faculty of Medicine, Yangzhou University, Yangzhou 225009, China.; 4 Department of Breast Surgery, The First Affiliated Hospital of China Medical University, Shenyang 110001, China.; 5 Jiangsu Co-innovation Center for Prevention and Control of Important Animal Infectious Diseases and Zoonosis, Yangzhou University, Yangzhou 225009, China.

**Keywords:** thyroid cancer, triptolide, GANT61, cell death, gasdermins, TAK1, cancer stem cells

## Abstract

Anaplastic thyroid cancer (ATC) is a lethal malignancy characterized by therapy resistance and rapid recurrence, driven in part by a population of thyroid cancer stem cells (CSCs). While the Gli1 inhibitor GANT61 targets CSC self-renewal, its efficacy is limited by compensatory activation of the pro-survival TAK1-AMPK pathway. Our present study aims to determine whether triptolide (TPL), a natural compound that inhibits TAK1 and induces reactive oxygen species (ROS), can overcome this resistance and achieve a synergistic anticancer effect when use with GANT61. Here, we report that TPL blocked GANT61-induced TAK1-AMPK activation. TPL alone effectively inhibited the proliferation of two ATC cell lines (8505C and SW1736) and achieved a synergistic anti-proliferative effect when used in combination with GANT61. Consistently, TPL alone effectively suppressed SW1736 xenograft tumor growth but, when used in combination with GANT61, prevented tumor recurrence. TPL in combination with GANT61 synergistically inhibited thyroid CSC self-renewal and induced apoptosis and pyroptosis. Mechanistically, TPL blocked GANT61-mediated activation of the TAK1-AMPK axis and synergistically induced ROS generation, which potentiated pyroptotic activity of Gasdermin D and E (GSDMD/E). This was confirmed by the evidence that TAK1 and AMPK inhibition by gene knockout or their specific inhibitors enhanced apoptotic and pyroptotic activity of GANT61, whereas ROS scavenge blocked apoptosis and pyroptosis induced by TPL and/or GANT61. Taken together, our study provides mechanistic insights into how TPL overcomes a key resistance pathway in CSC target therapy and suggests that the TPL-GANT61 combination could be a promising strategy to eradicate ATC.

## Introduction

Thyroid cancer is the most common malignancy of the endocrine system. Well differentiated papillary thyroid cancer (PTC), poorly differentiated thyroid cancer (DTC), and anaplastic thyroid cancer (ATC) represent 95%, 4%, and 1% of all cases, respectively 1. Majority of well differentiated PTCs can be cured through surgery and radioactive iodine treatment 2. BRAF-mutated PTCs exhibit reduced sensitivity to radioactive iodine, resulting in a poor overall prognosis 2. ATC is a rare and most aggressive malignancy characterized by its rapid progression and resistance to conventional therapies 3, 4. The median survival of ATC patients is about 9.5 months 3. Approximately 40% ATCs harbor a BRAF gene mutation 4, 5. Recent clinical trials revealed that BRAF/MEK inhibitors can rapidly shrink ATCs but the responses are short-lived 6. Given the limited efficacy of current therapies, there is urgent need in finding novel treatment modalities. Cabanillas et al. 7 recently proposed a “total therapy” approach which integrates targeted therapy plus immunotherapy, surgery, and radiation, with an attempt to eradicate all residual ATC cells. Novel approaches such as Ga-Mn bimetallic nanodecoy that induces intrinsic apoptosis and at mean time activates innate antitumor immunity may have therapeutic values for managing ATC 8.

Cancer stem cells (CSCs) are a subset of tumor cells with self-renewal capabilities. The Sonic Hedgehog (Shh) pathway activates the Gli1 transcription factor and plays a pivotal role in mediating the self-renewal of thyroid cancer stem cells 9, 10. ATC contains a higher percentage of CSCs than PTC 11. We reported earlier that the Shh pathway is highly activated in thyroid cancer and is required for maintaining thyroid CSC self-renewal 12. Thyroid CSCs also play an important role in drug resistance to the B-Raf kinase inhibitor vemurafenib 13. Intriguingly, thyroid cancer that have acquired adaptive resistance to vemurafenib contain fewer CSCs than their parental naïve controls 14. Inhibition of the Shh pathway by GANT61, a Gli1 inhibitor, blocks thyroid tumorigenesis initiated by thyroid cancer CSC but has low antitumor activity 10. Further investigation reveals that GANT61 activates the TAK1-AMPK-ULK1 axis by an unknow mechanism to induce autophagy and inhibit apoptosis 15. NF-κB is the downstream transcription factor readily activated by TAK1 16. NF-κB inhibits apoptosis by inducing the expression of cFLIP 17 and promotes tumor growth and CSC self-renewal 18. This may explain the poor response of ATC to GANT61 15. Additionally, TAK1 activation contributes to cell survival, angiogenesis, and metastasis by other mechanisms 16. There has been great interest in developing TAK1 inhibitors as novel anticancer drugs 16, 19.

Pyroptosis is a form of programmed cell death induced by pores formed by Gasdermin (GSDM) proteins in the plasma membrane 20. Pyroptosis induced by BRAF plus MEK inhibitors plays an important role in modulating immune cell populations and overcoming drug resistance in melanoma 21, 22. Induction of pyroptosis is considered a vital strategy for improving the efficacy of target therapy for thyroid cancer 23. Prosapogenin A (PA), a natural steroid saponin derived from *Veratrum nigrum L.*, exerts its anticancer activity against ATC by inducing pyroptosis 24. Ruxolitinib, a JAK-specific inhibitor, induces pyroptosis to suppress ATC growth *in vitro* and *in vivo* 25. There has been great interest in understanding the mechanisms of regulation of pyroptosis. Recent studies demonstrated that AMPK phosphorylates GSDMD and GSDME to inhibit their aggregation and pore formation 26, 27, whereas ROS induces GSDMD palmitoylation and activation even in the absence of GSDMD cleavage 28. Better understanding how anticancer agents induce pyroptosis will help design novel therapeutics for cancer treatment.

TPL is a bioactive ingredient extracted from *Tripterygium Wilfordii Hook F,* a Chinese traditional medicine that has been used as an anti-inflammatory and immunosuppressive remedy. TPL targets several cellular proteins such as TAB1 (TAK1-binding protein) 29, Prdx1/2 (Peroxiredoxin I/2) 30, 31, and RNA polymerase II 32. TPL possesses potent anticancer activities against a wide range of malignancies such as pancreatic, lung, and breast cancers by inducing apoptosis 33. TPL and its analogs increase reactive oxygen species (ROS) production, reduces Bcl-2 expression, impairs mitochondrial functions, and induces cell apoptosis by inhibiting the activity of NF-κB 33, 34. Whether TPL enhances pyroptosis by inhibiting AMPK and inducing ROS remains unclear. Our present study aims to determine the synergistic anticancer effects of GANT61 and TPL on ATC and understand the underlying mechanisms. Here, we report that TPL and GANT61 combination synergistically inhibits ATC cell growth and prevents tumor recurrence. Mechanistically, TPL in combination with GANT61 blocks GANT61-induced TAK1 and AMPK activation, synergistically induces ROS production, apoptosis, and pyroptosis, and restricts thyroid CSC self-renewal. Our study suggests that GANT61 and TPL combination therapy represents a novel combination strategy that targets thyroid CSCs and synergistically induces cell death to eradicate ATC.

## Materials and Methods

**Reagents and antibodies.** GANT61 was purchased from TargetMol Chemicals Inc. (Boston, MA). TPL was purchased from Sichuan Vicky Biotechnology Co., Ltd. (Chengdu, Sichuan, China). Compound C (CC) was purchased from Selleck Chemicals LLC (Shanghai, China) and dissolved in dimethyl sulfoxide (DMSO). 5Z-7-Oxozeaenol (5Z) and Z-VAD (OMe)-FMK (HY-16658) were purchased from Cayman Chemical (Ann Arbor, MI, USA) and MedChemExpress (Monmouth Junction, NJ, USA). TurboFect Transfection Reagent and the FastDigest Bmsb I restriction enzymes were purchased from New England Biolabs (Ipswich, MA, USA). pEASY®-Uni Seamless Cloning and Assembly Kit (#CU101-01) was purchased from Transgen (Beijing, China). Fluorescein (FITC) Annexin V Apoptosis Detection Kit I was purchased from BD Pharmingen (San Jose, CA, USA). Antibodies for TAK1 (phospho-Ser412) (#9339S), TAK1 (phospho-Thr187) (#4536), TAK1 (4505), AMPK (#phospho-Thr172) (#2535), AMPK (#5831), pp65 (phospho-Ser536) (#3033), Snail (#3895), BMI1 (#6964), caspase-3 (#9662), cleaved caspase-3 (#9664), caspase-8 (#4790), human cleaved caspase-8 (Asp374) (#9496), PARP (#9532), GSDMD (E9S1X) (#39754), JNK (phospho-Thr183/Tyr185) (#4668), JNK (#9252), and caspase-9 (#9508) were purchased from Cell Signaling Technology, Inc. (Danvers, MA). The antibody for SOX2 (#AM2048) was purchased from ABGENT, Inc. (San Diego, CA, USA). Antibodies against Gli1 (#sc-515751), p65 (#sc-372), Bcl-2 (#sc-7382), BAX (#sc-7480), β-Actin (#sc-47778), HMG-1/HMGB1 (sc-56698), and GAPDH (#166574) were obtained from Santa Cruz Biotechnology Inc. (San Diego, CA). The antibody for GSDME (#215191) was purchased from Abcam (Cambridge, MA, USA). The antibody for caspase-9 (66169-1-lg), Smac/DIABLO (68480-1-lg), and cFLIP (10394-1-AP) were obtained from Proteintech Group, Inc‌ (Wuhan, Hubei, China).

**Cells.** SW1736 cells (RRID: CVCL_3883) were kindly provided by Dr. Kenneth B. Ain 27, authenticated, and reported earlier 24. SW1736 cells were grown in complete RPMI 1640 media containing 10% fetal bovine serum. 8505C cells (RRID:CVCL_1054) were purchased from the Deutsche Sammlung von Mikroorganismen und Zellkulturen (DSMZ) (Science Campus Braunschweig-Süd, Germany) and were cultured in complete DMEM supplemented with 10% FBS. Both cell lines were negative for mycoplasma contamination examined by PCR.

**Gene knockout.** TAK1, GSDMD, and GSDME knockout was carried out by transfecting 8505C and SW1736 cells with a CRISPR expression vector encoding a guild RNA that targets TAK1, GSDMD, and GSDME. Each gene was targeted with three CRISPR constructs. The targeting sequences that successfully knocked out the expression of these three genes were as follows: TAK1, 5'-CACCGAGTTGTTTGCAAAGCTAAG-3' and 5'-AAACCTTAGCTTTGCAAACAACTC-3'; GSDMD, 5'-CACCGCCGCGGGGACAACGTGTACG-3' and 5'-AAACCGTACACGTTGTCCCCGCGGC-3'; GSDME, 5'-CACCGATGAAGACTGGCTCTCTACG-3' and 5'-AAACCGTAGAGAGCC-GTCTTCATC-3'. The complementary oligonucleotides containing 4 extra nucleotides at each end were annealed and ligated to the Bmsb I-digested LentiCRISPRv2 vector with the T4 DNA ligase. SW1736 and 8505C cells seeded in a 24-well plate were transfected with the LentiCRISPRv2 vector or the vector encoding sgRNA by using the TurboFect transfection reagent following the manufacturer's instruction. After incubation for 6 h, culture media were replaced with fresh complete media. Forty-eight hours later, single cell suspensions were prepared, seeded in 6-well plates, and grown in the media containing puromycin (0.5 or 0.75 μg/ml). Fresh media containing the same concentrations of puromycin were changed every three days. After culturing for 3-4 weeks, individual clones were picked, expanded, and analyzed for TAK1, GSDMD, and GSDME expression by Western blot. At least two clones for each knockout cell lines were selected and confirmed to give similar results.

**Cell proliferation assay.** SW1736 and 8505C cells were seeded in 96-well plates (2000 cells/well). After adherence, GANT61 and TPL were added at the indicated concentrations. After incubation for 72 h, cell proliferation was analyzed by using an ATP-based CellTiter-Glo kit (Promega). The IC_50_ values were calculated using GraphPad software (Fig. 1I & J). Data are presented as the mean ± standard deviation (SD) from triplicate wells, and the experiment was independently repeated twice with consistent results. To determine the synergistic effects of two compounds on cell proliferation (Fig. 1M & N), 8505C and SW1736 cells seeded in 96-well plates were incubated in the absence or presence of serially diluted GANT61 and TPL. Cell proliferation was conducted as above. Synergy scores were calculated to determine the synergistic effect of GANT61 and TPL on their cytotoxicity by using the SynergyFinder web tool based on the Highest Single Agent (HSA) model 35. A synergy score ≥10 indicates a synergistic effect, while a score between -10 and 10 indicates an additive effect (Fig. 1M &N). Alternatively, the cell proliferation of 8505C and SW1736 cells was similarly assessed using the Cell Counting Kit-8 (CCK-8; NCM Biotech, Suzhou, Jiangsu, China) (Fig. 1K & L, Fig. 9E & F)).

**Western Blot.** Cells grown in 12-well plates were harvested and lysed in NP-40 lysis buffer (50 mM Tris-HCl (pH 8.0), 150 mM NaCl, 1% NP-40, 5 mM EDTA, 10 mg/ml aprotinin). After incubation on ice for 30 min, cell lysates were prepared by spinning down at 4℃, 15,000 rpm for 15 min. After mixing with an equal volume of 2x loading buffer, samples were incubated at 95℃ for 5 min. Cell lysates were analyzed by Western blot with antibodies against the proteins of interest, followed by horseradish peroxidase-conjugated goat anti-rabbit or anti-mouse IgG and SuperSignal Western Pico enhanced chemiluminescence substrate (Pierce Chemical Co., Rockford, IL). The experiments were repeated at least three times with similar results. In case of two proteins of interest with close molecular masses, only one molecular marker was included in each blot. The density of the bands was analyzed by using NIH Image-J software and normalized by the arbitrary units of their corresponding total proteins, β-actin or GAPDH as indicated.

**Thyrosphere culture.** 8505C and SW1736 cells were incubated in the absence or presence of TPL (50 nM for 8505C, 25 nM for SW1736) for 48 h. Cells were trypsinized and counted and then seeded into a 24-well ultralow attachment plate (2000 cells/well) in serum-free DMEM/Ham's F-12 (1:1) medium containing B-27 (1:50) (Life Technology), basic fibroblast growth factor (bFGF), and epidermal growth factor (EGF) (20 ng/ml each). The numbers of thyrospheres were counted on day 14 and presented as the mean ± SD of three independent experiments.

**Cell death assay.** Wild-type and knockout SW1736 and 8505C cells seeded in 96-well plates were incubated with the various concentrations of TPL, GANT61, 5Z, and CC as recommended by manufacturers. After incubation for 48 h, the conditioned media were collected and analyzed for lactate dehydrogenase (LDH) activity by using a Cytotoxicity Detection Kit (Sigma-Aldrich, St. Louis, MO, USA) following the manufacturer's instruction (Fig. 4A & B, Fig. 6A, Fig. 7A & B, Fig. 8A, Fig. 9C & D). Data in bar graphs represents the mean ± SD of three independent experiments. Subsequently, the Bliss scores for each two-drug combination were calculated based on the Bliss independence model to assess synergistic or antagonistic interactions.

**Flow cytometry.** The effect of TPL on thyroid CSC renewal was investigated by flow cytometry analysis of aldehyde dehydrogenase (ALDH) positivity. Briefly, 8505C and SW1736 cells were incubated in the absence or present of TPL (50 nM for 8505C; 25 nM for SW1736) for 24 h. Single-cell suspensions were prepared and analyzed for ALDH activity by using an ALDEFLUOR kit (STEMCELL Technologies, Inc., Vancouver, BC, Canada) as previously reported 13. Diethylaminobenzaldehyde (DEAB), an ALDH-specific inhibitor, was added to the aliquot of 8505C and SW1736 cells as a background control. ALDH-positive cells were gated and statistically analyzed. Data in bar graphs are the mean ± SD of three independent experiments.

The effect of TPL and GANT61 on apoptosis was investigated by flow cytometric analysis of Annexin V and propidium iodide (PI) positivity. Briefly, SW1736 and 8505C cells seeded in six-well plates were incubated in the absence or presence of GANT61 (10 μM) minus or plus TPL (25 nM for SW1736; 50 nM for 8505C) for 24 h. Single-cell suspensions were prepared and stained with PI and Annexin V by using a FITC Annexin V Apoptosis Detection kit following the manufacturer's instructions. Cells were run in a Beckman Coulter flow cytometer (Model CyAn ADP). The fluorescence intensity was analyzed by using the CytExpert software. Annexin-positive and PI-positive cells were gated. The percentage of Annexin V- and PI-positive cells from three independent experiments were calculated and statistically analyzed by using the unpaired Student's *t* test. Data in bar graphs are the mean ± SD of three independent experiments.

**Pyroptosis analysis.** Wild-type and TAK1 or GSDMD&E knockout SW1736 and 8505C cells seeded in 6-well plates were incubated in the absence or presence of GANT61 (10 μM) minus or plus TPL (25 or 50 nM), 5Z (5 μM), or CC (10 μM) for 48 h. Static bright fields were randomly photographed under an Olympus microscope at 40x magnification. Cells with and without a bubbling morphology in each image were counted. Cells with bubble-like characteristics represent the number of cells undergoing pyroptosis in each field. The mean number of pyroptotic cells in five randomly selected fields were calculated. The mean number from three independent experiments were then pooled and statistically analyzed for significant differences between control group and treatment group.

**ROS analysis.** SW1736 and 8505C cells seeded in 6-well plates were incubated in the absence or presence of GANT61 (10 μM) and TPL (25 nM for SW1736; 50 nM for 8505C) alone or in combination for 48 h. Cells were stained with the fluorescent dye DCFH-DA (2,7-dichlorodihydrofluorescein diacetate) by using a Reactive Oxygen Species Assay Kit following the manufacturer's instructions. Images were captured using a Nikon or Olympus fluorescent microscope at 20x magnification. Single-cell suspensions were prepared and stained with DCFH-DA for intracellular ROS levels and analyzed in a Beckman Coulter flow cytometer (Model CyAn ADP) with CytExpert software.

**Subcellular fractionation of mitochondria and cytoplasm.** SW1736 and 8505C cells seeded in 6-well plates were incubated in the absence or presence of TPL and GANT61 alone or in combination for 48 h, the cells were harvested by trypsinization and washed once with ice-cold PBS. Subcellular fractionation of mitochondria and cytoplasm was performed using the Cell Mitochondria Isolation Kit (Beyotime, Shanghai, China) according to the manufacturer's instructions. Briefly, cell pellets were resuspended in ice-cold mitochondrial isolation reagent, homogenized on ice, and centrifuged at low speed to remove nuclei and unbroken cells. The resulting supernatant was then centrifuged to pellet the mitochondrial fraction. The supernatant from this final centrifugation was retained as the cytoplasmic (cytosolic) fraction. The fractions were analyzed for the levels of Smac by Western blot (Fig. 5G).

**Mice.** The use of animals was approved by the Institutional Animal Care and Use Committee of Yangzhou University. All experiments were conducted according to the Guide for the Care and Use of Laboratory Animals of the National Institutes of Health. The anticancer activity of TPL was first investigated in a xenograft model. Briefly, SW1736 cells were implanted subcutaneously into BALB/c *nu/nu* mice (5-6-wks-old; 5 mice per group; 8x10^6^ cells/mouse in 70 μl RPMI-1640 plus 30 μL Matrigel). When tumor volumes reached to approximately 400 mm^3^, the mice were treated with 0.5% DMSO as the vehicle control in 100 μl PBS or TPL (0.6 mg/kg every other day) by intraperitoneal injection for 16 days (Fig. 2A-E). The tumor growth inhibition rate was calculated using the formula: {1- (the mean tumor volume of the TPL group at the end of treatment ÷ the mean tumor volume at the beginning of the TPL group) divided by (the mean tumor volume of the control group at the end of treatment ÷ the mean tumor volume at the beginning of the control group) x 100%. To examine the synergistic anticancer effects of GANT61 and TPL, SW1736 cells were implanted subcutaneously into BALB/c nu/nu mice as above. When tumor volumes reached to approximately 400 mm^3^, mice were randomly divided into four groups in a blinding fashion: the vehicle control (ethanol:corn oil, 1:4 vol:vol; 4 mice/group), GANT61 (50 mg/kg every other day; 4 mice/group), TPL (0.2 mg/kg/day; 8 mice/group), TPL plus GANT61 groups (8 mice/group). The exclusion criteria were to make sure that the mean tumor volumes in four groups were similar. Mice in these four groups were first treated for two weeks as illustrated in Figure 2F. Mice in the control and GANT61-treated group were sacrificed due to the volume of tumor reached to the size stipulated by the NIH guidelines. Mice in the TPL and TPL plus GANT61 groups were rested for two weeks and then treated with the same dose for another two weeks. Tumor volumes were measured three times per week with a vernier caliper and calculated based on the formula: length x width^2^ x 0.5. The bodyweights were recorded simultaneously. Tumor nodules were collected and used to prepare for single-cell suspensions followed by flow cytometric analysis of ALDH positivity. Mice were euthanized by cervical dislocation. One portion of tumor tissues from each mouse was lysed in NP-40 lysis buffer at a weight/volume ratio of 1:15 and homogenized. The resulting cell lysates were subjected to Western blot analysis to detect the expression of indicated proteins.

**Statistical analysis.** For statistical analysis, all data are presented as mean ± standard deviation (SD) from at least three independent experiments unless otherwise specified. Comparisons between two groups were performed using unpaired two-tailed Student's *t* test. For comparisons involving three or more groups under a single independent variable, one-way analysis of variance (ANOVA) was used, followed by Tukey's multiple comparisons test to compare all possible pairs of groups. For experiments involving two independent variables (e.g. treatment × genotype, treatment × NAC, or treatment × time), two-way ANOVA was applied, followed by Šídák's multiple comparisons test for pre-planned simple-effect comparisons, which included: (i) comparisons among different treatment groups within the same genotype or NAC condition, and (ii) comparisons between genotypes or NAC conditions within the same treatment group. For tumor xenograft studies with repeated measurements over time (tumor volume and body weight), two-way ANOVA was performed with Šídák's correction. The *p* value of < 0.05 was considered statistically significant. All analyses were conducted using GraphPad Prism software (Version 8.0.2 or later).

## Results

**TPL blocks GANT61-mediated TAK1 and AMPK phosphorylation and synergistically inhibit ATC cell proliferation.** We first examined the impact of GANT61 and TPL alone or in combination on TAK1 and AMPK phosphorylation. GANT61 dose- and time-dependently induced the phosphorylation of TAK1 and its two downstream substrates, AMPK and the p65 subunit of NF-κB in SW1736 and 8505C cells, two BRAF-mutated anaplastic thyroid cancer cell lines (Fig. 1A & B), and in WRO82 cells, a follicular thyroid carcinoma cell line with wild-type BRAF Fig. S1A). TPL binds TAB1 to inhibit TAK1 activation in LPS- and TNF-stimulated cells (29. TPL dose- and time-dependently inhibited TAK1^S412^ and TAK1^T187^ phosphorylation in SW1736 and 8505C cells (Fig. 1C & D) and WRO82 cells Fig. S1B). TPL blocked GANT61-induced TAK1, AMPK, and p65 phosphorylation in these two cell lines (Fig. 1E - H) and in WRO82 cells (Fig. S1C). TPL and GANT61 alone dose-dependently inhibited the proliferation of 8505C, SW1736 (Fig. 1I & J) and WRO82 cells (Fig. S1D). The IC_50_ values of TPL for 8505C and SW1736 cells were 33.6 nM and 14.1 nM (Fig. 1I & J), respectively. The IC_50_ values of GANT61 for 8505C and SW1736 cells were 12.7 and 8.4 μM, respectively (Fig. 1I & J). The IC_50_ values of TPL and GANT61 for WRO82 cells were 51.8 nM and 6.4 μM (Fig. S1D), respectively. Combination of TPL (3.75 nM) and GANT61 (10 μM) exhibited a synergistic anti-proliferative effect in 8505C cells (Fig. 1K) (Combination index (CI) = 0.817). Combination of TPL and GANT61 at other concentrations exhibited an additive anti-proliferative effect in 8505C and SW1736 cells (Fig. 1K & L) (CI values between 0.8-1.0). The synergistic cytotoxic effect of TPL and GANT61 combination was confirmed by two systemic dose-dose matrix experiments with the Bliss Independence model. The synergy scores for TPL and GANT61 combination on SW1736 and 8505C cells are 10.83 and 9.97, respectively (Fig. 1M & N). Combination of TPL and GANT61 gained only additive cytotoxic effect on WRO82 cells (the synergy score of 7.12) (Fig. S1E).

**TPL and GANT61 synergistically inhibited SW1736 xenograft growth and prevents recurrence**. We next examined the antitumor activity of TPL alone and in combination with GANT61 in a xenograft model. TPL treatment (0.6 mg/kg per other day) prevented the growth of SW1736 xenograft implanted subcutaneously in Babl/c *nu/nu* mice (Fig. 2A & B). The tumor growth inhibition rate (TGI) reached 70.7%, indicating significant suppression of tumor growth. TPL treatment did not cause bodyweight changes during the two-week treatment period (Fig. 2C). Although TPL used at 1 mg/kg/day causes significantly hepatoxicity and nephrotoxicity, when used at 0.3 mg/kg/day, it does not increase aspartate transaminase (AST), alanine transaminase (ATL), creatinine, and BUN levels (36. TPL treatment decreased the levels of the stemness-related genes including Gli1, SOX2, BMI1, Nanog, and Oct4 (Fig. 2D) and decreased the number of ALDH-positive cells in the SW1736 xenograft tissues (Fig. 2E). When the volumes of SW1736 xenografts reached to the approximately 400 mm^3^, mice were treated with TPL and GANT61 alone or in combination for 2 weeks (Fig. 2F). Again, TPL and GANT61 alone or in combination did not affect the bodyweights, compared to the vehicle control (Fig. 2G). GANT61 weakly but significantly slowed down the growth of SW1736 xenografts, with a TGI of 19.87% (Fig. 2H). Since this observation is in line with our previous finding 10, only four mice in the control and GANT61 groups were used. TPL plus GANT61 (TGI=87.22%) shrank xenografts more effectively than TPL alone (TGI=78.66%) (Fig. 2H). To determine the effect of TPL and GAN61 combination in preventing tumor recurrence, mice treated with TPL or TPL plus GANT61 underwent the second cycle treatment (Fig. 2I & J). TPL and GANT61 combination prevented the regrowth of SW1736 xenografts (Fig. 2I). However, xenografts in mice treated with TPL alone rapidly regrew after the second cycle treatment (Fig. 2I & J). Four of eight xenografts in mice treated with TPL plus GANT61 were eradicated (Fig. 2J). Western blot analysis revealed that TPL alone or in combination with GANT61 significantly reduced the levels of the stemness-related genes in the xenograft tissues, compared to control samples in untreated mice (Fig. 2K). These observations collectively suggest that TPL in combination with GANT61 has synergistic effects on the expression of the stemness-related genes and on thyroid tumor regrowth. Though TPL and GANT61 had opposite effect on the activation of the TAK1-AMPK axis (Fig. 1A-D), the synergistical effect of TPL and GANT61 *in vivo* on tumor regrowth is in part due to their inhibitory effect on stemness-related gene expression.

**TPL and GANT61 synergistically inhibit the expression of the stemness-related genes.** The Shh pathway plays an important role in maintaining thyroid CSC self-renewal 10, 12, 37, 38. NF-κB upregulates the expression of the stemness-related genes such as SOX2 and Gli1 39. TPL directly binds HNF1, a transcription factor that inhibits the expression of several genes in the Shh pathway including Shh, Gli2, and PTCH in non-small cell lung cancer 40. To examine the synergistic effect of GANT61 and TPL on thyroid CSC self-renewal, we first examined their inhibitory effect on the expression of the stemness-related genes. Both GANT61 and TPL inhibited the expression of the stemness-related genes including SOX2, Gli1, BMI1, and Snail in 8505C and SW1736 cells in a dose- and time-dependent manner (Fig. 3A & B). GANT61 in combination with TPL further reduced the levels of these stemness-related gene expression in 8505C and SW1736 cells at 24 h (Fig. 3C). TPL treatment significantly decreased the percentage of ALDH-positive cells (Fig. 3D-G) and the numbers of thyrospheres (Fig. 3H-I) in 8505C and SW1736 cells.

**TPL and GANT61 combination synergistically induces apoptosis and pyroptosis.** AMPK phosphorylates GSDME at Thr6 and Ser46 to inhibit caspase-3-mediated GSDME cleavage and activation, leading to inhibition of pyroptosis 27. Since GANT61 activates TAK1 and AMPK 15, we hypothesized that blockade of the TAK1-AMPK axis by TPL may sensitize thyroid cancer cells to GANT61-induced cell death. GANT61 or TPL alone weakly or modestly increased the levels of LDH in the conditioned media of SW1736 and 8505C cells, respectively (Fig. 4A & B). To determine if the effect of GANT61 plus TPL on LDH release was additive or synergistic, we calculated the predicted and observed index of two drug combination by using the Bliss independence model and found that the observed index (0.86) in the TPL (50 nM) plus GANT61 (10 μM) combination is larger than the predicted index (0.69). This suggests that TPL and GANT61 act synergistically to induce LDH release, a marker of cell death (Fig. 4A). TPL induced the cleavage of caspase-3, caspase-8, PARP, GSDMD, and GSDME much more potently than GANT61 (Fig. 4C & D). GANT61 in combination with TPL further increased the cleavage of caspase-3, caspase-8, PARP, GSDMD, and GSDME than GANT61 or TP alone (Fig. 4C & D). TPL and GANT61 in combination significantly increased the levels of HMGB1 and GAPDH in the conditioned media of 8505C and SW1736 cells (Fig. 4D), which serves as a marker to indicate pyroptosis 41.

GANT61 induced apoptosis in approximately 10% of 8505C and SW1736 cells (Fig. 4E-H). TPL induced apoptosis in approximately 25% of 8505C cells and 17% of SW1736 cells (Fig. 4E-H). GANT61 plus TPL induced apoptosis in approximately 38% of 8505C and 25% of SW1736 cells (Fig. 4E-H). 8505C and SW1736 cells treated with GANT61 or TPL alone or in combination presented with the swelling and bubble-like morphology (Fig. 4I & K), which is one of the major characteristics of pyroptosis 42. However, very few SW1736 and 8505C cells treated with GANT61 alone underwent pyroptosis (Fig 4I & K). The numbers of pyroptotic 8505C and SW1736 cells treated with GANT61 plus TPL were much higher than that treated with TPL or GANT61 alone (Fig. 4J & L).

Finally, we confirmed the role of caspases in cleaving GSDMD and GSDME in TPL- and GANT61-treated 8505C and SW1736 cells pretreated with the pan-caspase inhibitor Z-VAD. Z-VAD profoundly suppressed the cleavage of GSDME (Fig. 4M) in these two cell lines treated with TPL and/or GANT61. TPL plus GANT61 weakly induced GSDMD cleavage in both cell lines, which were also blocked by Z-VAD. These observations collectively suggest that caspase activation is essential for GSDMD/E processing induced by TPL and GANT61 in these two cell lines.

**ROS contributes to TPL- and GANT61-induced apoptosis and pyroptosis.** ROS plays an important role in oxidizing and damaging DNA and mitochondrial membrane proteins, causing the release of the cytochrome C from the mitochondria, activation of caspase-9, and intrinsic apoptosis 43. Enhancing ROS production and immunological cell death by using an anisotropic gold-palladium heterostructured nanosystem or by using the pH-responsive neutrophil membrane camouflage Ga-Mn bimetallic nanodecoy can greatly potentiate the therapeutic effects of radioimmunotherapy or metal ion-based biomimetic nanomedicine, respectively 8, 44. Furthermore, a recent study indicates that ROS induces GSDMD palmitoylation, lysosomal leakage, and pyroptosis in a caspase-3-independent manner 28. We then examined the effect of GANT61 and TPL on ROS production, apoptosis, and pyroptosis. TPL increased the levels of ROS more potently than GANT61 in 8505C and SW1736 cells (Fig. 5A-D). TPL in combination with GANT61 further increased the levels of ROS in these two cell lines (Fig. 5B & D). ROS has been implicated in activating JNK 45. Both GANT61 and TPL alone induced JNK phosphorylation in a time- and dose-dependent manner in these two cell lines (Fig. 5E-F). GANT61 in combination with TPL increased JNK phosphorylation at a higher magnitude in 8505C and SW1736 cells than that treated with GANT61 or TPL alone (Fig. 5G). GANT61 alone weakly or modestly increased both Bcl-2 and Bax expression (Fig. 5G). TPL modestly inhibited Bcl-2 expression but slightly increased Bax expression (Fig. 5G). GANT61 in combination with TPL decreased Bcl-2 and increased Bax expression efficiently (Fig. 5G). However, GANT61 and TPL alone or in combination had little or weak effects on caspase-9 activation, respectively (Fig. 5G). The combined use of GANT61 and TPL slightly increased caspase-9 cleavage, significantly decreased the levels of cFLIP but increased the release of Smac in the cytoplasm of 8505C and SW1736 cells, compared to that treated with GANT61 or TPL alone (Fig. 5G). The ROS scavenger N-acetylcysteine (NAC) inhibited TPL- and/or GANT61-induced caspase-8, caspase-3, PARP, GSDMD, and GSDME cleavage, and increased the Bcl-2/Bax ratio (Fig. 5H). Consistent with these observations, NAC also decreased the number of pyroptotic 8505C and SW1736 cells treated with GANT61 and TPL alone or in combination (Fig. 5I-L).

**TAK1 inhibition enhances GANT61-induced cell death.** To determine if TPL sensitized thyroid cancer to GANT61 by inhibiting TAK1, we first tested if TAK1 inhibition by 5Z-7- Oxozeaenol (5Z), a TAK1-specific inhibitor, enhanced GANT61-induced cell death. GANT61 and 5Z alone significantly increased the levels of LDH released into the conditioned media of SW1736 and 8505C cells (Fig. 6A). GANT61 in combination with 5Z further increased the LDH release into the conditioned media of these two cell lines, compared to that treated with GANT61 or 5Z alone (Fig. 6A). A relatively small number of SW1736 and 8505C cells underwent pyroptosis following 5Z treatment (Fig. 6B-E). However, GANT61 in combination with 5Z significantly increased the number of pyroptotic cells, compared to that treated with 5Z or GANT61 alone (Fig. 6B-E). Consistently, 5Z inhibited GANT61-induced TAK1 phosphorylation in 8505C and SW1736 cells and promoted the cleavage of GSDME, GSDMD, caspase-3, caspase-8, and PARP (Fig. 6F).

We next examined the effect of TAK1 knockout in GANT61- and TPL-induced apoptosis and pyroptosis. GANT61 and TPL alone or in combination increased the release of LDH into the conditional media of TAK1-deficient 8505C and SW1736 cells much more effectively than in wild-type control cells (Fig. 7A & B). In line with these observations, genetic ablation of TAK1 significantly enhanced pyroptosis in 8505C and SW1736 cells upon treatment with GANT61 but had little effect on TPL-induced pyroptosis (Fig. 7C & D). Moreover, TAK1 knockout blocked GANT61-induced AMPK phosphorylation but increased the cleavage of caspase-8, PARP, and GSDME in 8505C and SW1736 cells treated with GAN61 and/or TPL (Fig. 7E & F). Unexpectedly, TAK1 knockout blocked GSDMD cleavage in 8505C and SW1736 cells treated with TPL or TPL plus GANT61 (Fig. 7E & F). The absence of the N-terminal pore-forming domain of GSDMD in TAK knockout cells treated with TPL and/or is likely due to its cleavage by caspase-3, which cleaves human GSDMD at D87 and inactivates it 46.

**AMPK inhibition enhances GANT61-induced cell death.** Given that AMPK phosphorylation of GSDMD and GSDME suppresses their oligomerization and pyroptosis 26, 27, we next investigated whether AMPK inhibition could also potentiate GANT61-induced cytotoxicity. The AMPK inhibitor Compound C (CC, 5 μM) itself significantly increased LDH release (Fig. 8A) and pyroptotic cell numbers in SW1736 and 8505C cells, which was further enhanced by GANT61 (Fig. 8B-E). CC abrogated GANT61-induced AMPK phosphorylation and promoted the cleavage of GSDME, GSDMD, caspase-3, caspase-8, and PARP (Fig. 8F).

**Pyroptosis contributes to TPL- and GANT61-induced cell death.** TPL induces pyroptosis in head-and-neck cancer 47. Here we tested if pyroptosis contributed to the cytotoxic effect of GANT61 and TPL. GSDMD and GSDME were successfully knocked out by two CRISPR constructs (Fig. 9A & B). GSDMD/E knockout slightly or moderately increased the levels of PARP and caspase-8 cleavage in 8505C and SW1736 cells treated with TPL and/or GANT61 L (Fig. 9A & B). GSDMD/E knockout significantly inhibited the release of LDH in these two cell lines treated with TPL alone or TPL plus GANT61, compared to that in wild-type cells (Fig. 9C & D). GSDMD/E knockout significantly improved the viability in these two cell lines treated with GANT61 or/and TPL (Fig. 9E & F). GANT61 and TPL alone or in combination were no longer able to induce pyroptosis in GSDMD/E knockout SW1736 and 8505C cells (Fig. 9G-J). These observations suggest that pyroptosis contributes to GANT61- and TPL-induced cell death.

## Discussion

TPL targets multiple cellular proteins and exerts its cytotoxic and anticancer activity mainly by inducing cell death. Our present study provides evidence that TPL in combination with GANT61 synergically activates caspase-8/3 and GSDMD/E to induce apoptosis and pyroptosis, suppresses the expression of the stemness-related genes and thyroid CSC self-renewal, and prevents tumor re-growth. Mechanistically, TPL blocked TAK1 and AMPK activation; TPL in combination with GANT61 synergistically induced ROS production and JNK activation by blocking GANT61-activated TAK1; Pyroptosis was required for TPL- and GANT61-induced cell death. Our study provides novel insights into the mechanisms of TPL-induced cell death and suggests that TPL in combination with GANT61 holds great potential for effectively treating anaplastic thyroid cancer.

The mechanisms of TPL-induced apoptosis are complex and remain incompletely understood. TPL inhibits the expression of HSP70, a 70-kDa chaperone that plays a crucial role for protein folding. HSP70 downregulation leads to ER stress and apoptosis 32, 48. TPL inhibits NF-κB activation and Bcl-2 expression 32. Decreased Bcl-2 expression compromises mitochondrial membrane potential and induces the cytochrome C release, which activates Apaf and caspase-9 and its downstream caspase-3 49. TPL induces caspase-9 activation in the HL60 leukemia cell line 50. Although TPL inhibits Bcl-2 expression and induces mitochondrial apoptosis in Sertoli cells, gastric cancer cells, and the cytochrome C is readily released into the cytoplasm, caspase-9 is poorly activated or its activation is not shown in these studies 51, 52. Consistently, our present study showed that, TPL had little effect on caspase-9 activation (Fig. 5G), suggesting that caspase-9 plays a minor role in TPL-induced apoptosis in ATC. The inability of TPL to effectively activate caspase-9 in two ATC cell lines may reflect the intrinsic unique feature of ATC, which is highly insensitive to chemotherapy. However, other studies show that TPL used at 100 nM readily induces caspase-9 activation in nasopharyngeal carcinoma cells and cholangiocarcinoma cells 53, 54. Why TPL differentially activates caspase-9 in different types of malignancies remains unclear.

Over the past decade, the TNF receptor (TNFR)-mediated cell death has been well characterized 55. Upon TNF binding, TNFR initiates the formation of the complex I, which consists of the TRADD, TRAF2, cIAPs, and RIPK1 56, 57. cIAP ubiquitinates RIPK1 to activate TAK1 and its downstream MAP kinase pathways and NF-κB, which then induces the transcription of Bcl-2, cFLIP, and cIAPs 56, 57. Smac, a protein released from mitochondria due to JNK-induced tBid cleavage 58, disrupts the interaction of TRAF2 and cIAP and induces cIAP degradation 56, 57. Consistently, our present study shows that TPL activated JNK to induce mitochondrial Smac release into the cytoplasm and inhibited NF-κB activation to suppress cFLIP expression (Fig. 5G). This leads to the dissociation of the RIPK1-FADD-caspase-8 complex from TNFR, caspase-8 activation, and apoptosis (Fig. 10). GANT61 had a weak cytotoxic effect on thyroid tumor cells that is likely caused by ROS production and subsequent apoptosis. In addition, GANT61 also has a cytostatic activity on thyroid tumor cells caused by inhibition of the Shh pathway 12. 5Z and TAK1 knockout modestly or weakly increased GANT61-induced apoptosis, the levels of GANT61-induced apoptosis in TAK1-deficient cells were much lower than TPL in wild-type cells. This suggests an additional action such as decreased cFLIP expression is needed for GANT61 to effectively induce apoptosis.

Emerging evidence suggests that anticancer drugs exert their cytotoxic and antitumor effects not only by inducing apoptosis but also by pyroptosis. For example, commonly prescribed anticancer drugs such as doxorubicin, erlotinib, and trametinib induce concurrent apoptosis and pyroptosis via caspase-8/3-activated GSDMD/E in some cancer cell contexts 59-61. Alantolactone (ATL), a terpenoid extracted from traditional Chinese medicinal herb *Inula helenium L*., induces concurrent apoptosis and GSDME-dependent pyroptosis of anaplastic thyroid cancer through ROS mitochondria-dependent caspase pathway 62. TPL induces GSDME-mediated pyroptosis in head and neck cancer by suppressing the expression of c-Myc and mitochondrial hexokinase-II 47. Our present study showed that TPL induced GSDMD/E cleavage, acetylcysteine (NAC) inhibited TPL-induced caspase-8/3 and GSDMD/E activation. These observations suggest that TPL-induced pyroptosis is mediated by caspase-8/3-activated GSDMD/E. Ruxolitinib, a JAK-specific inhibitor, induces apoptosis and GSDMD/E-mediated pyroptosis in anaplastic thyroid cancer by inhibiting DRP1-mediated mitochondrial fission 25.

Several recent studies indicate that GSDMD/E posttranslational modification regulates their activation. For example, AMPK phosphorylates GSDMD and GSDME to inhibit their aggregation and pore formation 26, 27. ROS induces GSDMD palmitoylation and activation even in the absence of GSDMD cleavage 28. Based on these observations, we speculate that induction of ROS and inhibition of AMPK by TPL may enhance GSDMD/E activation by palmitoylating GSDMD and by inhibiting GSDMD/E phosphorylation 63. GSDMD/E knockout abrogated TPL-induced pyroptosis, reduced TPL-mediated cytotoxic activity, and improved the viability of TPL-treated SW1736 and 8505C cells. This suggests that TPL induces cell death not only by inducing apoptosis but also by pyroptosis. In support of this notion, Jiang et al. reported that GSDME-induced pyroptosis sensitizes the cytotoxic and anticancer activity of 5-FU on colon cancer cells *in vitro* and *in vivo* 64. Prosapogenin A, a bioactive ingredient prevalent in traditional Chinese herbs, exerts its cytotoxic and antitumor effects on anaplastic thyroid cancer by inducing pyroptosis through ATPase-mediated lysosomal over-acidification 24. Cisplatin-induced pyroptosis via the NLRP3/caspase-1/GSDMD pyroptosis pathway contributes to its anticancer activity against triple negative breast cancer 65. These observations collectively suggest that TPL and chemotherapeutic drugs may exert their anticancer activity by potentiating caspase-D/E-mediated pyroptosis.

It is well established that NF-κB is involved in maintaining the characteristics of cancer stem cells and plays a crucial role in tumorigenesis, development, and metastasis 18. For example, NF-κB activation by IRAK1 leads to CSC enrichment and confers resistance to paclitaxel in triple negative breast cancer 66, 67. Consistent with these observations, our present study showed that TPL inhibited the phosphorylation of the p65 subunit of NF-κB and the expression of the stemness-related genes in two anaplastic thyroid cancer cell lines. In addition, TPL may inhibit the expression of the stemness-related genes by binding and inhibiting the transcriptional activity of HNF1A, a transcription factor that is involved in the transcription of the stemness-related genes 40. Our present study showed that TPL blocked GANT61-activated TAK1 and synergistically blocked the expression of thyroid CSC-related genes. Our prior studies have shown that activation of the Sonic hedgehog pathway plays a crucial role in thyroid CSC self-renewal, inhibition of the Gli1 transcription factor in the Shh pathway by GANT61 blocks thyroid CSC self-renewal 10. However, due to the activation of TAK1 and induction of autophagy, GANT61 has limited anticancer activity 15. GANT61 in combination with TPL partially eradicated the established ATC xenografts in immunodeficient mice. These findings collectively suggest that inhibition of the expression of the stemness-related genes by TPL and GANT61 by different mechanisms may have synergistic effects on blocking CSC-mediated tumor recurrence and metastasis.

The poor water solubility and high toxicity of TPL limit its clinical application 32. In particular, TPL causes cytotoxicity in many noncancerous cell lines such as human kidney HK-2 cells or rat heart H9c2 cells and primary rat cardiomyocytes, aortic smooth muscle cells, Sertoli cells, and granulosa cells with the IC_50_ values equivalent or lower than that in tumor cells 36. TPL also has a potent toxicity *in vivo* in experimental animals 36. Xu et al. 68 reported that the LD_50_ of TPL in mice is 0.83 mg/kg. Mice administered with 2 mg/kg or 1 mg/kg TPL all die on second day or on day 7, respectively 36. Preclinical studies indicate that the *in vivo* toxic effect of TPL is mainly on liver, kidney, heart, hematological, neurological, reproductive, and digestive systems 69, 70. While TPL has potential therapeutic values for autoimmune-related thyroid diseases, it does not seem to affect the normal thyroid function 69, 70. One approach to lowering its toxicity is to synthesize the analogs of TPL with better solubility and lower toxicity 32, 71. For example, Tian et al. 34 recently reported that the TPL analog CK21 induces ROS production and inhibits NF-κB activation and the growth of pancreatic cancer in a xenograft mouse model. Alternatively, the ligand-guided delivery and pH-sensitive nanoparticles have been tested for reducing the toxicity of TPL 32, 72. Alternatively, the toxicity of TPL could be reduced by combination with another anticancer cancer drug 32. For example, TPL in combination with paclitaxel and gemcitabine exhibited significantly lower toxicity and better antitumor activity against pancreatic cancer than paclitaxel plus gemcitabine chemotherapy alone 73. ATC is one of the most aggressive malignancies and almost fatal [3,4]. TPL in the form of the traditional Chinese medicine has been approved for the treatment of psoriasis, rheumatic, and inflammatory diseases 32. GANT61 has been widely used in preclinical studies for a variety of cancers 74-76. GANT61 does not cause significant hematological, renal and hepatotoxicity in a HeLa xenograft nude mouse model 77. GANT61 and TPL combination therapy may possess great therapeutic values in treating ATC.

In summary, our present study shows that TPL alone or in combination with GANT61 induces apoptosis and pyroptosis in two ATC cell lines. TPL induces apoptosis by activating the ROS-JNK axis and by inhibiting TAK1 and NF-κB activity (Fig. 10). TPL inhibits the expression of the stemness-related genes and blocks GANT61-activated TAK and AMPK to achieve a synergistical cytotoxic and antitumor effect in vitro and in vivo (Fig. 10). Our study provides novel mechanistic insights into the action of TPL and suggests that TPL and GANT61 combination may represent a novel therapeutic approach for treating anaplastic thyroid cancer.

## Supplementary Material

Supplementary figures.

## Figures and Tables

**Figure 1:**
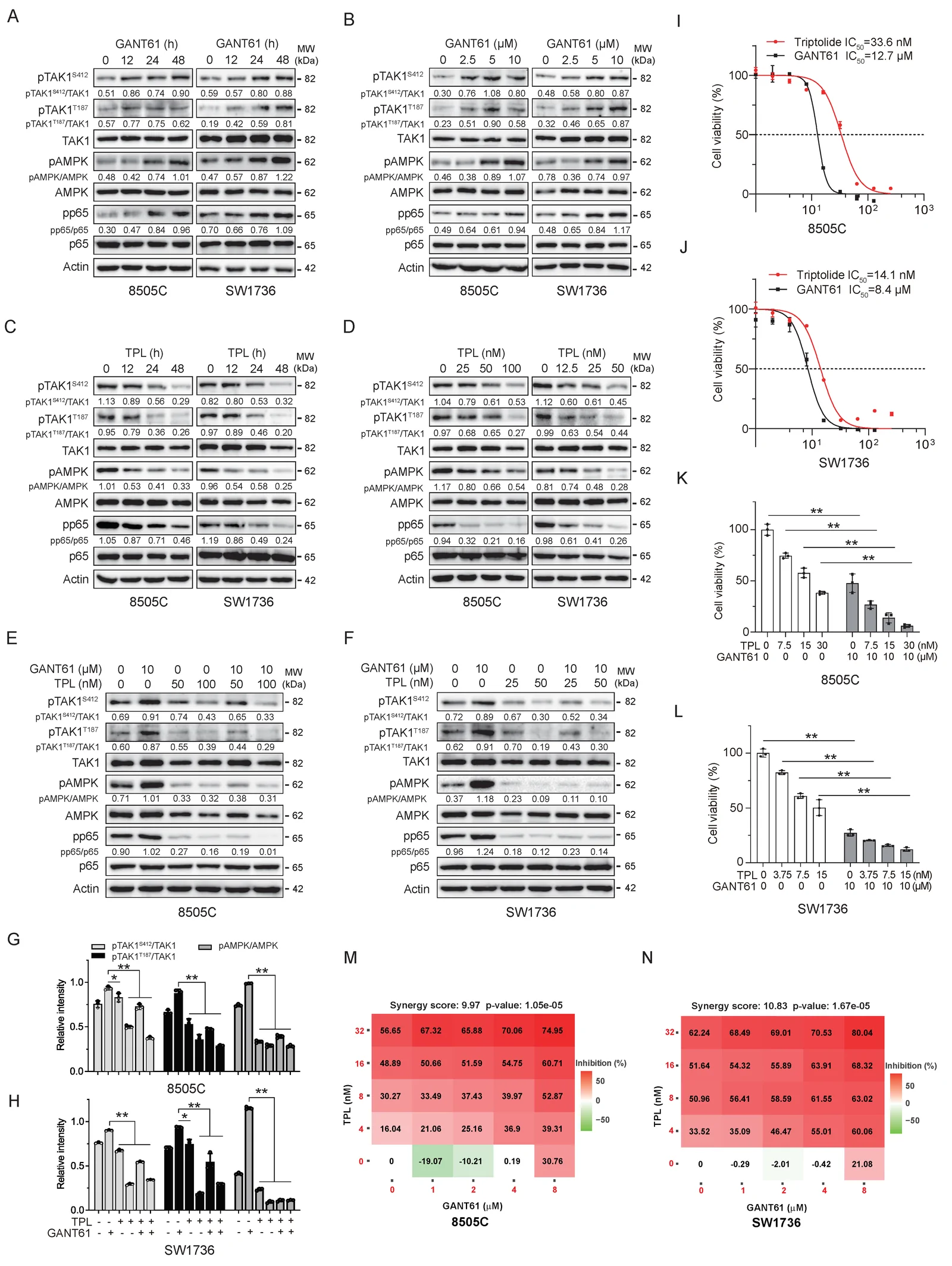
** The effect of TPL and GANT61 on TAK1 phosphorylation and cell proliferation.** (**A-D**) 8505C and SW1736 cells seeded in 12-well plates were treated with GANT61 (10 μM) or TPL (50 nM for 8505C, 25 nM for SW1736) for the indicated lengths of time or with the indicated concentrations of GANT61 or TPL for 48 h. (**E & F**) 8505C cells and SW1736 cells were incubated in the absence or presence of GANT61 (10 μM) minus or plus TPL (25, 50 or 100 nM) for 48 h. Cell lysates were prepared and analyzed for phosphorylation of TAK1^S412^, phospho-TAK1^T187^, AMPK, p65 as well as their total corresponding proteins by Western blot. The results represent one of three independent experiments. (G & H) Densitometric analysis of phosphorylated TAK1 (Ser412/Thr187) and AMPK levels. Band intensities were normalized to their respective total protein levels, and data are presented as mean ± SD from three independent experiments. Statistical significance was determined by one-way ANOVA with Tukey's multiple comparisons test. (**I & J**) 8505C and SW1736 cells seeded in a 96-well plate (2000 cells/well) were incubated with the indicated concentrations of TPL or GANT61 for 72 h. Cell proliferation was determined using a CellTiter-Glo kit. The IC_50_ values represent the mean ± standard deviation (SD) of three independent experiments, each in triplicate. (**K & L**) 8505C and SW1736 cells were incubated with the indicated concentrations of TPL minus or plus GANT61 (10 μM) for 72 h. Cell proliferation was determined by using a CCK8 kit. Data represents the mean ± SD of three independent experiments in a bar graph. Statistical significance was determined by two-way ANOVA with Sidak's multiple comparisons test. (**M & N**) Isobologram of GANT61 and TPL combination in 8505C and SW1736 cells. Cells (2,000/well) were seeded in 96-well plates and exposed to serial dilutions of GANT61, TPL, or fixed-ratio combinations for 72 h, followed by CellTiter-Glo viability assay. Synergy scores were calculated to quantify the drug-drug interaction, with positive scores indicating synergy. *p < 0.05, **p < 0.01, compared to the untreated control.

**Figure 2:**
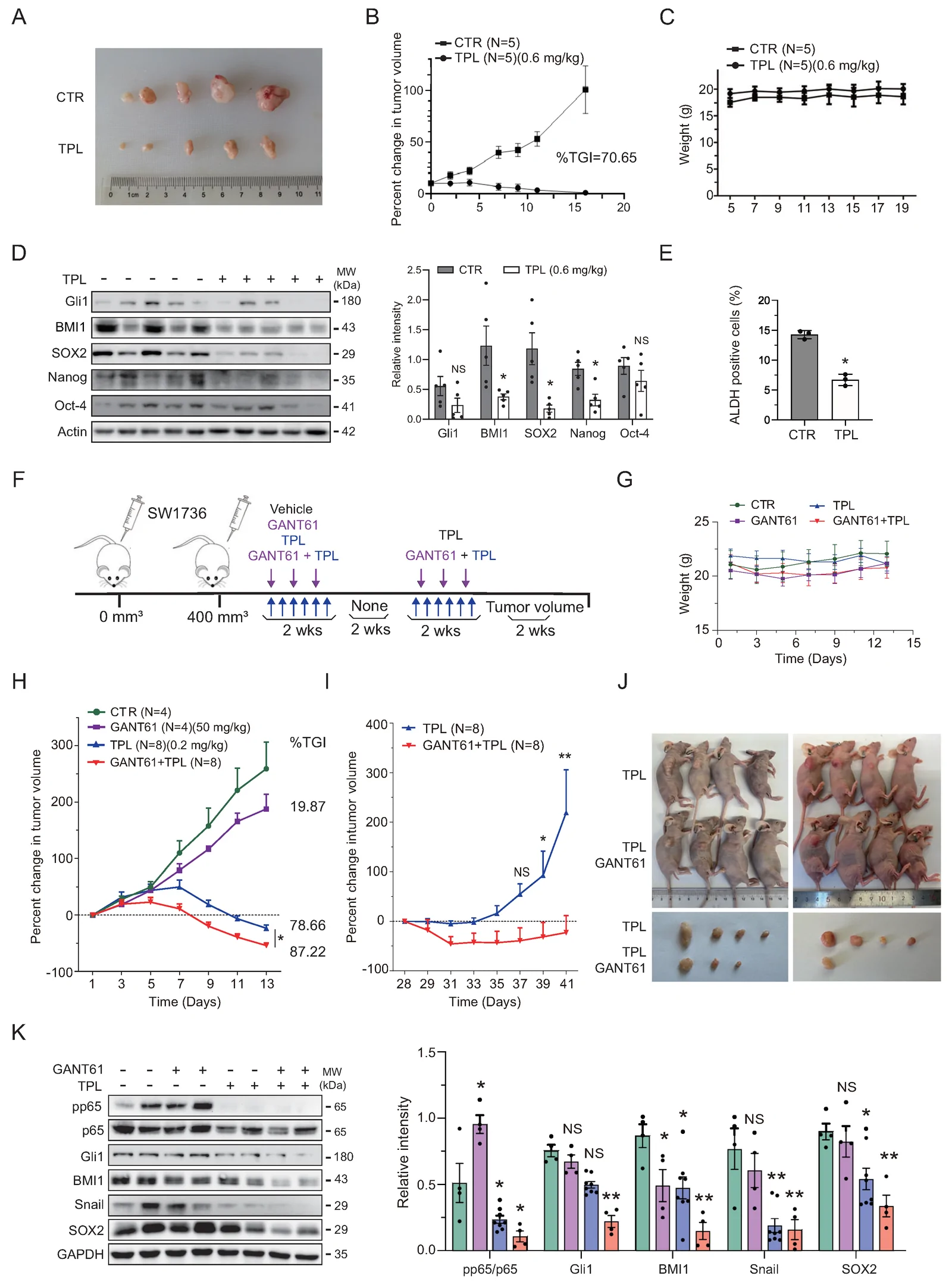
** GANT61 and TPL in combination synergistically inhibit xenograft growth.** (**A-E**) SW1736 cells (8x10^6^ cells/mouse) were injected subcutaneously into BALB/c *nu/nu* mice. When tumor volumes reached to approximately 400 mm^3^, the mice were treated for 16 days with 0.5% DMSO as the vehicle or TPL (0.6 mg/kg every other day, 5 mice per group) by intraperitoneal injection (**A**). Tumor volume was measured three times a week (**B**). TGI, the tumor growth inhibition rate. Mouse bodyweights were measured every other day **(C)**. (**D**) Tumor tissues were collected and analyzed for the expression of stemness-related genes by Western blot with the indicated antibodies. The relative protein levels were analyzed by quantifying the density of protein bands using NIH Image-J software and normalized to the density of the β-actin band as a loading control. The results represent the mean ± SD of tissue lysates from five mice per group. * p < 0.05; NS, not significant. (**E**) Single-cell suspensions from tumor tissues were analyzed for ALDH expression by flow cytometric analysis. Data are the mean ± SD of 5 xenografts. *p < 0.05. (**F-L**) SW1736 cells (8x10^6^ cells/mouse) were injected subcutaneously into 24 BALB/c *nu/nu* mice**.** When tumor volumes reached to approximately 400 mm^3^, mice were randomly divided into four groups. Four mice each were treated with corn oil as the vehicle control (ethanol:corn oil, 1:4 vol:vol) or with GANT61 (50 mg/kg every other day) for two weeks. Eight mice each were treated with TPL (0.2 mg/kg/d) or TPL plus GANT61 by intraperitoneal injection for two weeks (**F**). Mice were weighed (**G**) and monitored for tumor growth (**H**) every other day. Mice in TPL and TPL plus GANT61 groups were then left untreated for two weeks and then re-treated with the same doses of TPL or TPL plus GANT61 for another two weeks (**F**). Changes in tumor volumes were recorded and graphed (**I**). The two-way ANOVA with Šídák's multiple comparisons test was used to determine if the differences in tumor growth were statistically significant (**H & I**). *p < 0.05; **p < 0.01; NS, not significant. Tumor xenografts were harvested and photographed (**J**). Tumor tissue lysates were analyzed for the expression of stemness-related genes (**K**) by Western blot with the indicated antibodies. The relative levels of Gli1, BMI1, Snail, and SOX2 to GAPDH and the relative levels of p65 phosphorylation to its total protein were semi-quantified using NIH ImageJ software and presented as bar graphs. The results represent the mean ± SD of tissue lysates from four or eight mice per group. *p < 0.05; ** *p* < 0.01; NS, not significant.

**Figure 3:**
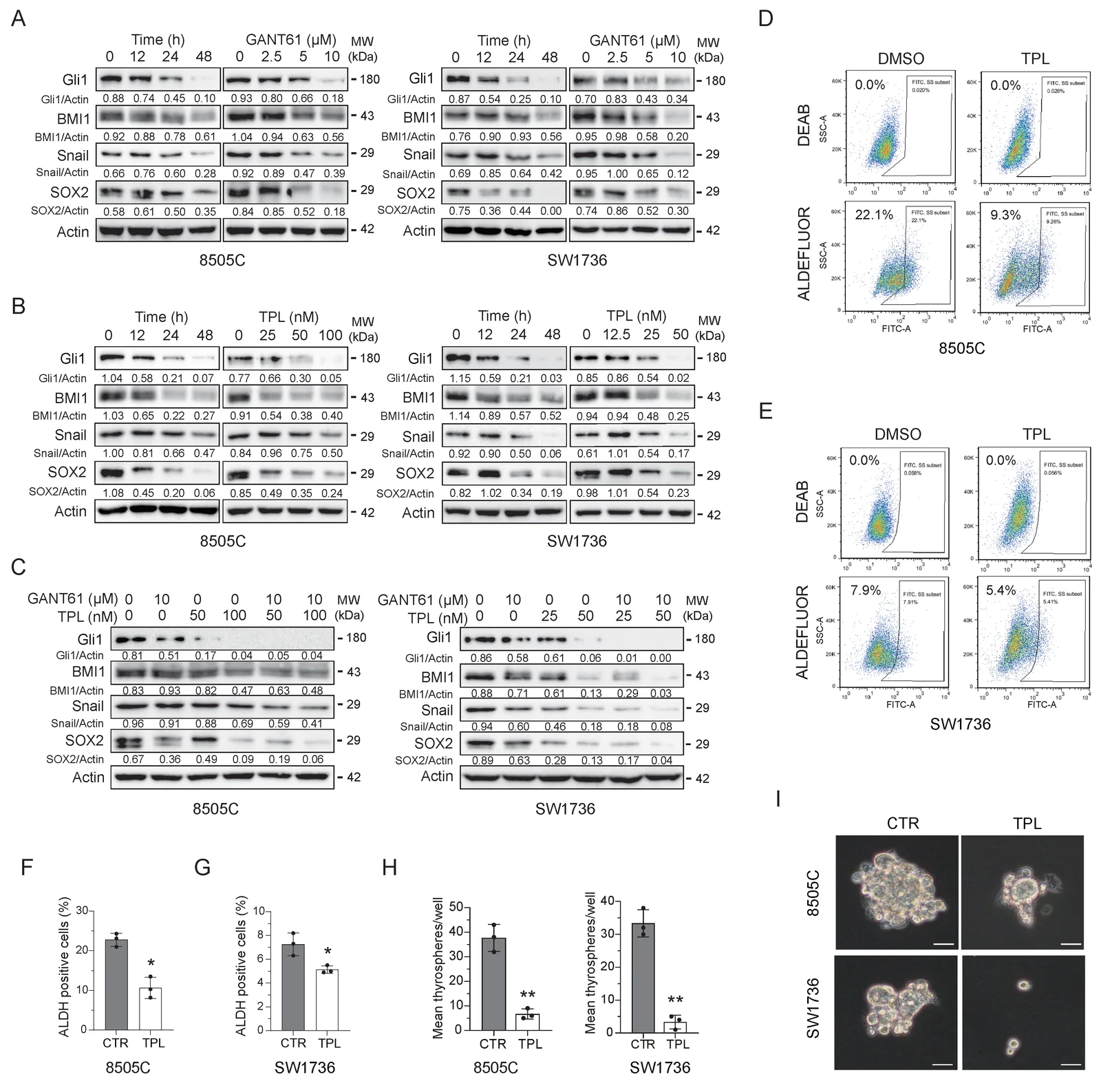
** TPL and GANT61 synergistically inhibit the expression of stemness-related genes.** (**A & B**) 8505C and SW1736 cells were treated with GANT61 (10 μM) or TPL (100 nM for 8505C, 50 nM for SW1736) for the indicated lengths of time or with the indicated concentrations of GANT61 (0, 2,5, 5, 10 μM) or TPL (0, 12.5, 25, 50 or 100 nM) for 48 h. Cell lysates were prepared and analyzed for Gli1, BMI1, Snail, SOX2, and Actin by Western blot. (**C**) 8505C and SW1736 cells were treated with GANT61 (10 μM) or the indicated concentrations of TPL for 24 h. Cell lysates were prepared and analyzed for Gli1, BMI1, Snail, SOX2, and Actin by Western blot. (**D-G**) 8505C and SW1736 cells were incubated for 24 h in the absence or presence of TPL (50 nM for 8505C; 25 nM for SW1736), respectively. Single-cell suspensions of 8505C and SW1736 cells were prepared and analyzed for ALDH positivity by flow cytometry. The ALDH inhibitor DEAB was included as the background control (**D & E**). The mean percentage of ALDH-positive cells in three independent experiments were calculated and plotted in bar graphs (**F & G**). *p < 0.05. **(H & I)** 8505C and SW1736 cells were incubated for 24 h in the absence or presence of TPL (50 nM for 8505C; 25 nM for SW1736), respectively. Single-cell suspensions of 8505C and SW1736 cells were cultured in the stem cell media in a 6-well ultralow attachment plate. Thyrospheres were photographed and enumerated on day 14. Data are the mean ± SD of three independent experiments. Scale bar, 50 μm. ** p < 0.01, compared to the control.

**Figure 4:**
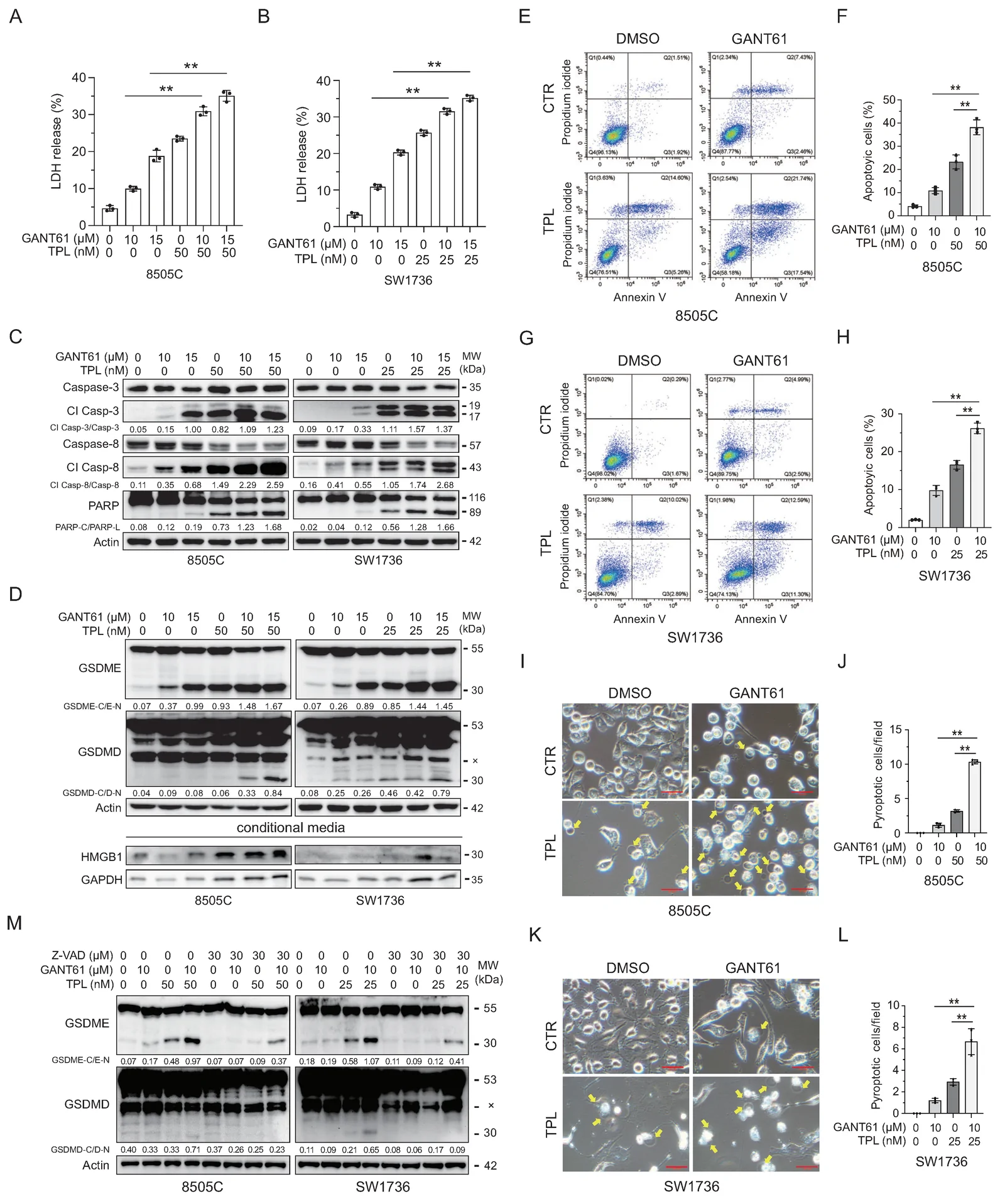
** TPL enhances GANT61-induced apoptosis and pyroptosis.** (**A & B**) 8505C and SW1736 cells were incubated in the absence or presence of GANT61 minus or plus TPL for 48 h. The conditioned media were collected and analyzed for cell death by measuring LDH activity. Data are the mean ± SD of three independent experiments. **p < 0.01, compared to the untreated control. (**C & D**) 8505C and SW1736 cells were incubated in the absence or presence of GANT61 minus or plus TPL for 48 h. Cell lysates were analyzed for the levels of caspase-3, cleaved caspase-3 (Cl-casp-3), caspase-8, cleaved caspase-8 (Cl-Casp-8), and PARP (**C**) or GSDME and GSDMD (**D**) by Western blot. × A nonspecific protein. Analysis of HMGB1 and GAPDH levels in cell culture medium using methanol-chloroform extraction protein method. (**E-H**) 8505C and SW1736 cells were incubated in the absence or presence of GANT61 (10 μM) minus or plus TPL (50 nM for 8505C, 25 nM for SW1736) for 24 h. Single-cell suspensions were stained for propidium iodide (PI) and Annexin V. The results represent the mean ± SD of three independent experiments (**G & H**). (**I-L**) 8505C and SW1736 cells treated with TPL and/or GANT61 as above were examined under an Olympus microscope at 40× magnification. Static bright fields were randomly photographed. Scale bar, 50 μm. The number of bubbling cells per field from five randomly selected fields were counted and statistically analyzed (**J & L**). (M) 8505C and SW1736 cells were pretreated with or without the pan-caspase inhibitor Z-VAD (30 μM) for 6 h. The cells were then treated with GANT61 and/or TPL for 48 h. Cell lysates were analyzed for GSDMD and GSDME cleavage by Western blot. Data represent the mean ± SD of three independent experiments. ** p < 0.01, compared to the control.

**Figure 5:**
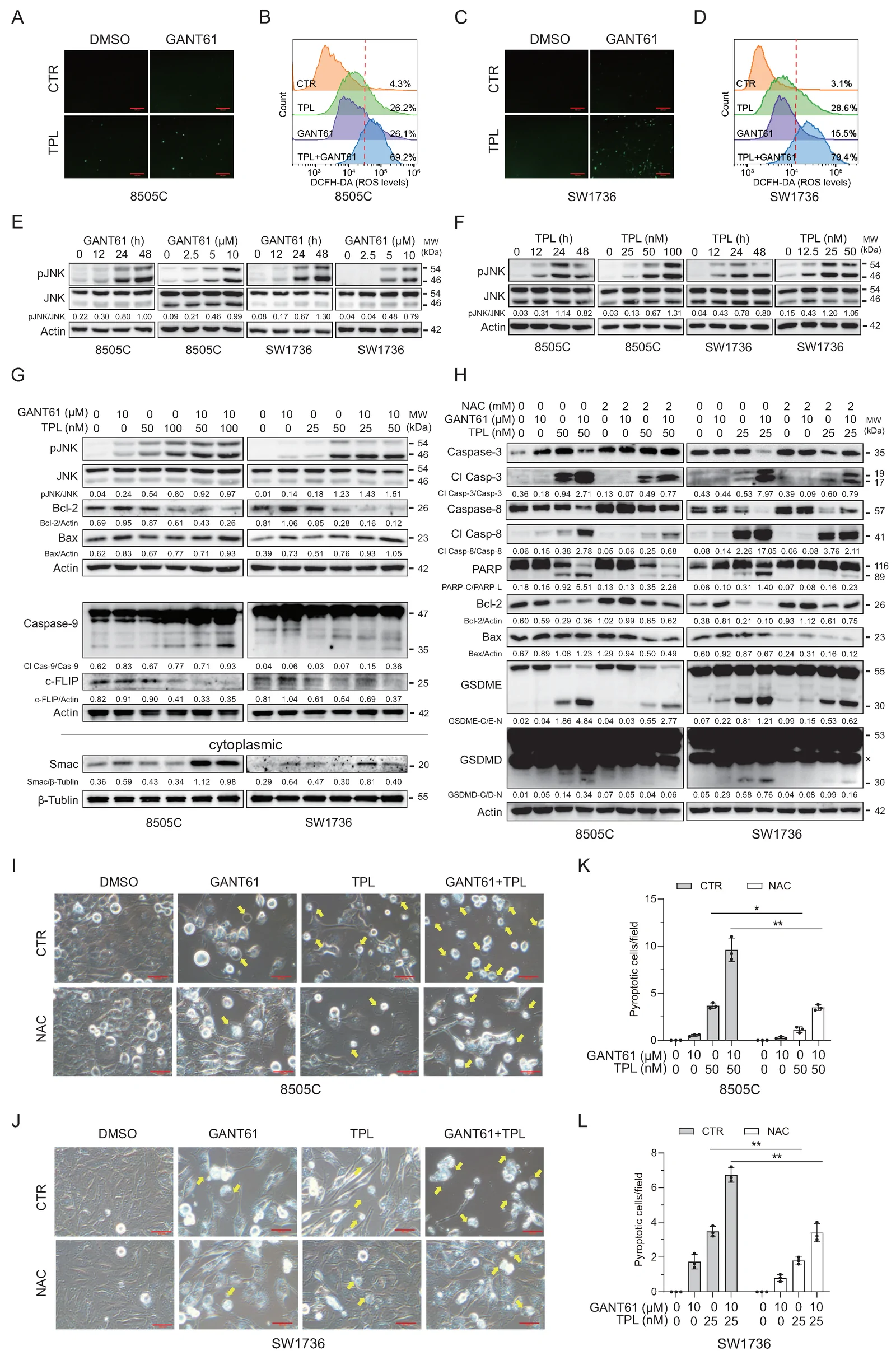
** GANT61- and TPL-induced ROS activates JNK and contributes to apoptosis.** (**A-D**) 8505C and SW1736 cells were treated in the absence or presence of GANT61 (10 μM) minus or plus TPL (50 nM for 8505C, 25 nM for SW1736) for 48 h. The cells were stained with the DCFH-DA fluorescent dye and examined under a fluorescent microscope (A & C). Alternatively, the levels of ROS in single-cell suspensions were stained with DCFH-DA and quantified in a flow cytometer (**B & D**). Data represent one of three independent experiments with similar results. (**E-G**) 8505C and SW1736 cells were treated with GANT61 (10 μM) or TPL (100 nM for 8505C, 50 nM for SW1736) for the indicated lengths of time or with the indicated concentrations of GANT61 and/or TPL for 48 h. Cell lysates were prepared and analyzed for the levels of phospho-JNK, JNK, caspase-9, Bcl-2, Bax, and c-FLIP by Western blot. The expression of Smac was detected in the cytoplasm of cells without mitochondria (G). (**H**) 8505C and SW1736 cells were incubated in the absence or presence of NAC minus or plus GANT61 and/or TPL for 48 h. Cell lysates were analyzed for the levels of caspase-3, cleaved caspase-3 (Cl Casp-3), caspase-8, cleaved caspase-8, PARP, Bcl-2, Bax, GSDME and GSDMD by Western blot. × A nonspecific protein. (**I-L**) 8505C and SW1736 cells were treated as above and analyzed for pyroptosis under a bright field microscope. Static bright fields were randomly photographed. Scale bar, 50 μm. (**K & L**) The number of bubbling cells was counted and statistically analyzed. Data represent the mean ± SD of three independent experiments. *p < 0.05; **p < 0.01.

**Figure 6:**
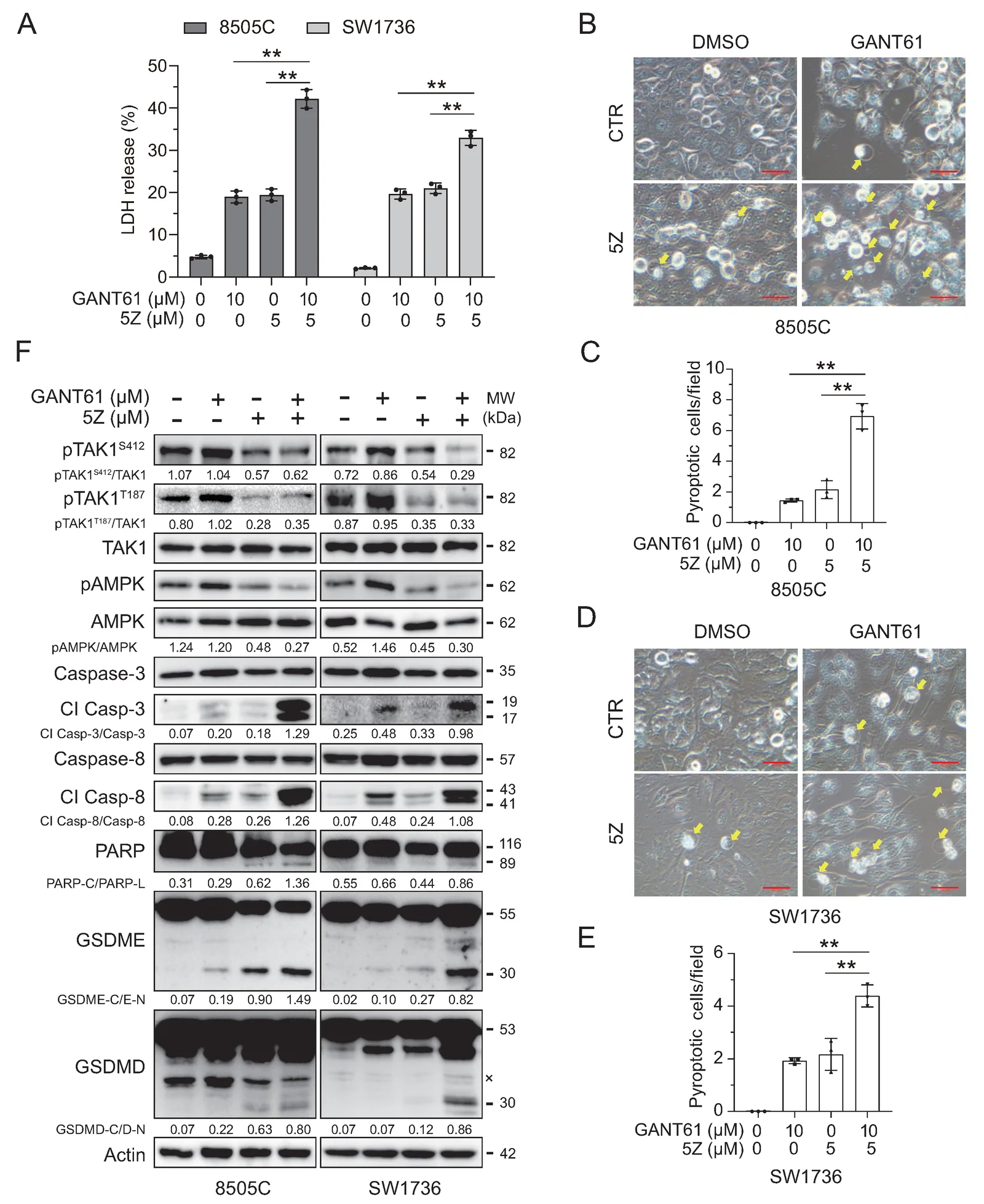
** TAK1 inhibition enhances GANT61-induced cell death.** (**A**) 8505C and SW1736 cells were incubated in the absence or presence of GANT61 minus or plus 5Z for 48 h. The conditioned media were collected and analyzed for cell death by measuring LDH activity. Data are the mean ± SD of three independent experiments. ** p < 0.01. (**B-E**) 8505C and SW1736 cells were treated as above and analyzed for pyroptosis under an Olympus microscope static bright field at 40× magnification were randomly photographed (**B & D**). Scale bar, 50 μm. The number of bubbling cells was counted and statistically analyzed (**C & E**). Data represent the mean ± SD of three independent experiments. ** p < 0.01. (**F**) 8505C and SW1736 cells were treated as above. Cell lysates were analyzed for the levels of indicated proteins by Western blot. Cl Casp-3, cleaved caspase-3; Cl Casp-8, cleaved caspase-8. × A nonspecific protein. The results represent one of three experiments with similar results.

**Figure 7:**
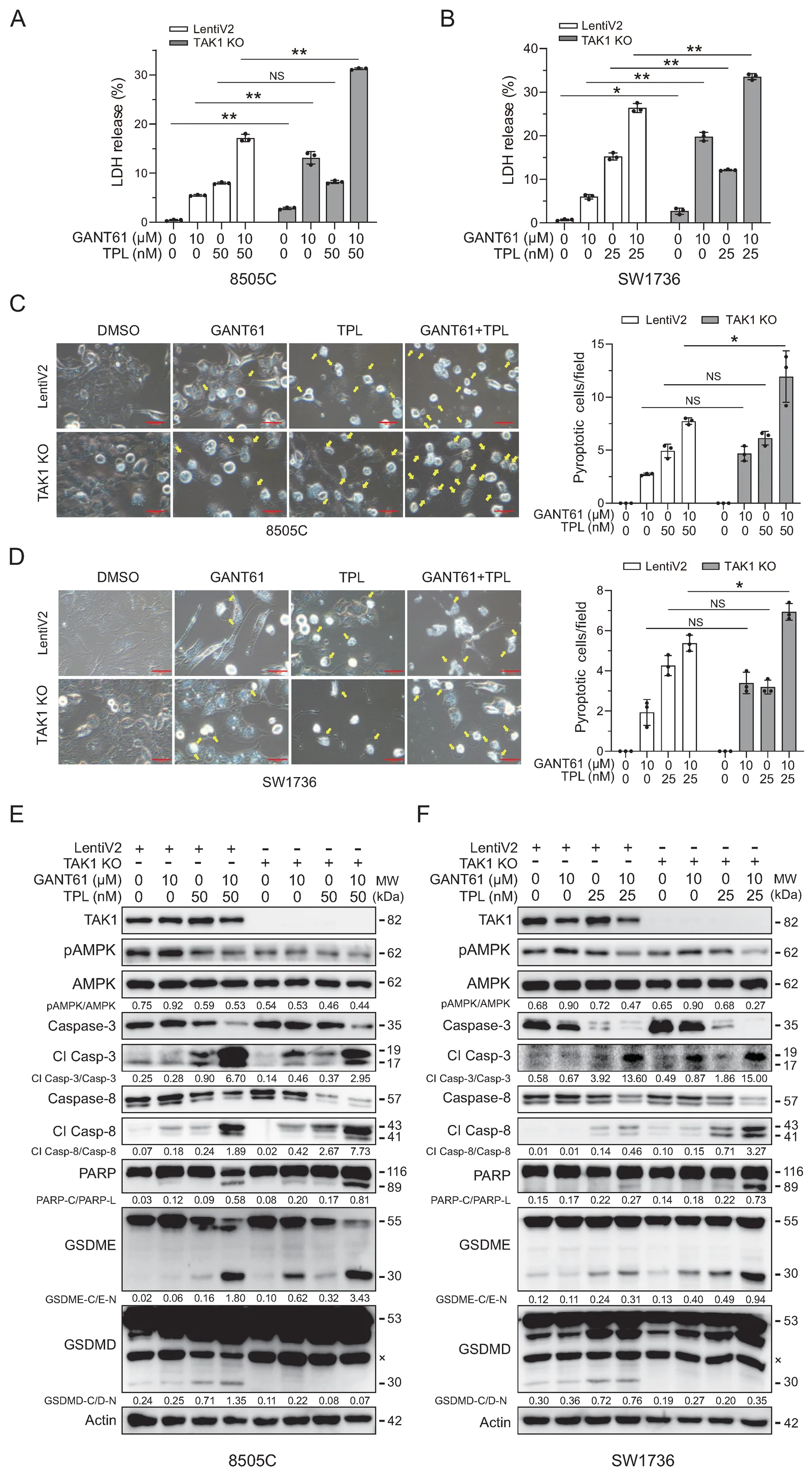
** TAK1 knockout enhanced TPL-induced cell death.** (**A & B**) Wild-type and TAK1 knockout 8505C and SW1736 cells were incubated in the absence or presence of GANT61 minus or plus TPL for 48 h. The conditioned media were collected and analyzed for cell death by measuring LDH activity. Data are the mean ± SD of three independent experiments. KO, knockout. (**C & D**) Control and TAK1 knockout 8505C or SW1736 cells were treated in the absence or presence of GANT61 minus or plus TPL for 48 h. Static bright fields were randomly photographed. Scale bar, 50 μm. Bubbling cells were counted and statistically analyzed. Data represent the mean ± SD of three independent experiments. *p < 0.05; ** *p* < 0.01; NS, not significant. (**E & F**) 8505C and SW1736 cells were treated as above. Cell lysates were prepared and analyzed for the indicated proteins by Western blot. The results represent one of three experiments with similar results. Cl Casp-3, cleaved caspase-3; Cl Casp-8, cleaved caspase-8. × A nonspecific protein.

**Figure 8:**
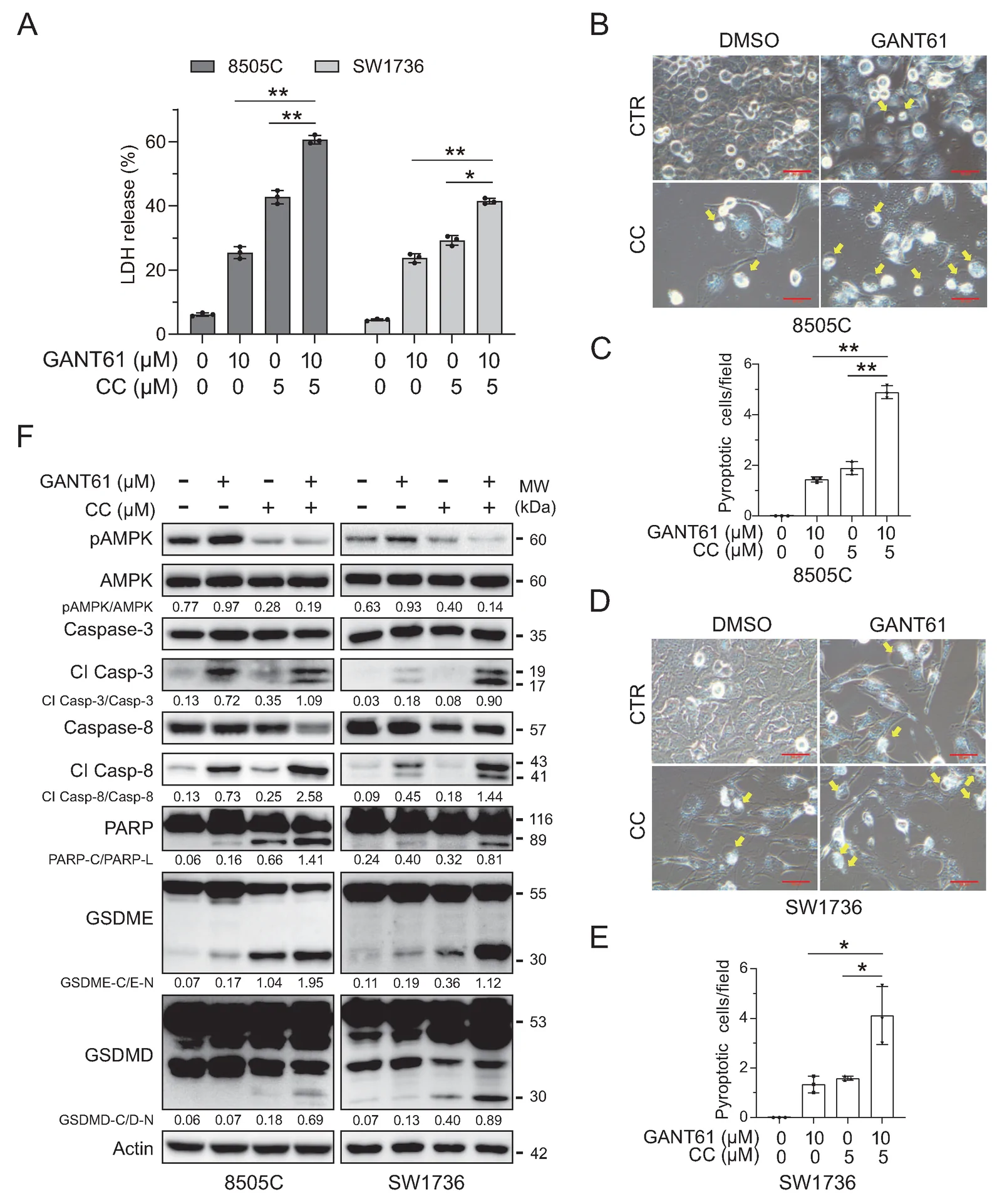
** AMPK inhibition enhances GANT61-induced cell death.** (**A**) 8505C and SW1736 cells were incubated in the absence or presence of GANT61 minus or plus compound C (CC) for 48 h. The conditioned media were collected and analyzed for cell death by measuring LDH activity. Data are the mean ± SD of three independent experiments. * *p* <0.05, ** p < 0.01. (**B-E**) 8505C and SW1736 cells were treated as above. Pyroptosis was examined under an Olympus microscope at 40× magnification. Scale bar, 50 μm. Bubbling cells were counted calculated and statistically analyzed. Data represent the mean ± SD of three independent experiments. *p < 0.05; **p < 0.01. (**F**) 8505C and SW1736 cells were treated as above. Cell lysates were prepared and analyzed the levels of the indicated proteins by Western blot. The results represent one of three experiments with similar results. Cl Casp-3, cleaved caspase-3; Cl Casp-8, cleaved caspase-8.

**Figure 9:**
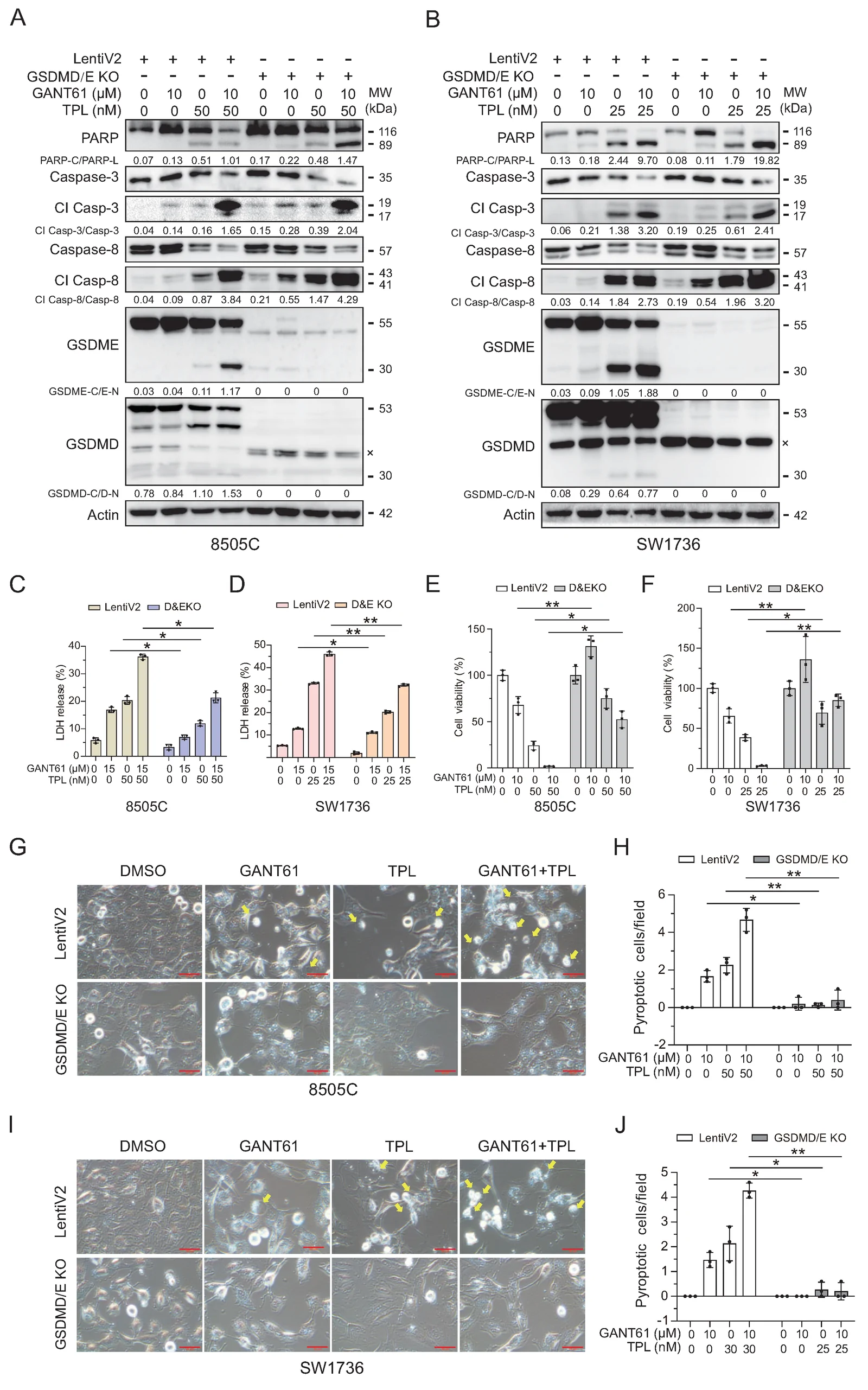
** Pyroptosis contributes to TPL and GANT61-induced cell death.** (**A & B**) Control and GSDMD/E knockout 8505C and SW1736 cells were incubated in the absence or presence of GANT61 (10 μM) minus or plus TPL (50 or 25 nM) for 48 h. Cell lysates were analyzed for the expression of the indicated proteins by western blot. Cl Casp-3, cleaved caspase-3; Cl Casp-8, cleaved caspase-8. KO, knockout. × A nonspecific protein. (**C & D**) The conditioned media collected from the cells treated in the absence or presence of GANT61 (15 μM) minus or plus TPL (50 or 25 nM) for 48 h were analyzed for cell death by measuring LDH activity. Data are the mean ± SD of three independent experiments. (**E & F**) Control and GSDMD&E knockout cells seeded in a 96-well plate (2000 cells/well) were incubated with the indicated concentrations of GANT61 (10 μM) minus or plus TPL (50 or 25 nM) for 72 h. Cell proliferation was determined using a CCK8 kit. Data represents the mean ± SD of three independent experiments in a bar graph. (**G-J**) Control and GSDMD/E knockout 8505C and SW1736 cells were treated as above. Pyroptosis was examined under an Olympus microscope at 40× magnification. Scale bar, 50 μm. Bubbling cells were counted calculated and statistically analyzed (**H & J**). Data represent the mean ± SD of three independent experiments. *p < 0.05; **p < 0.01.

**Figure 10:**
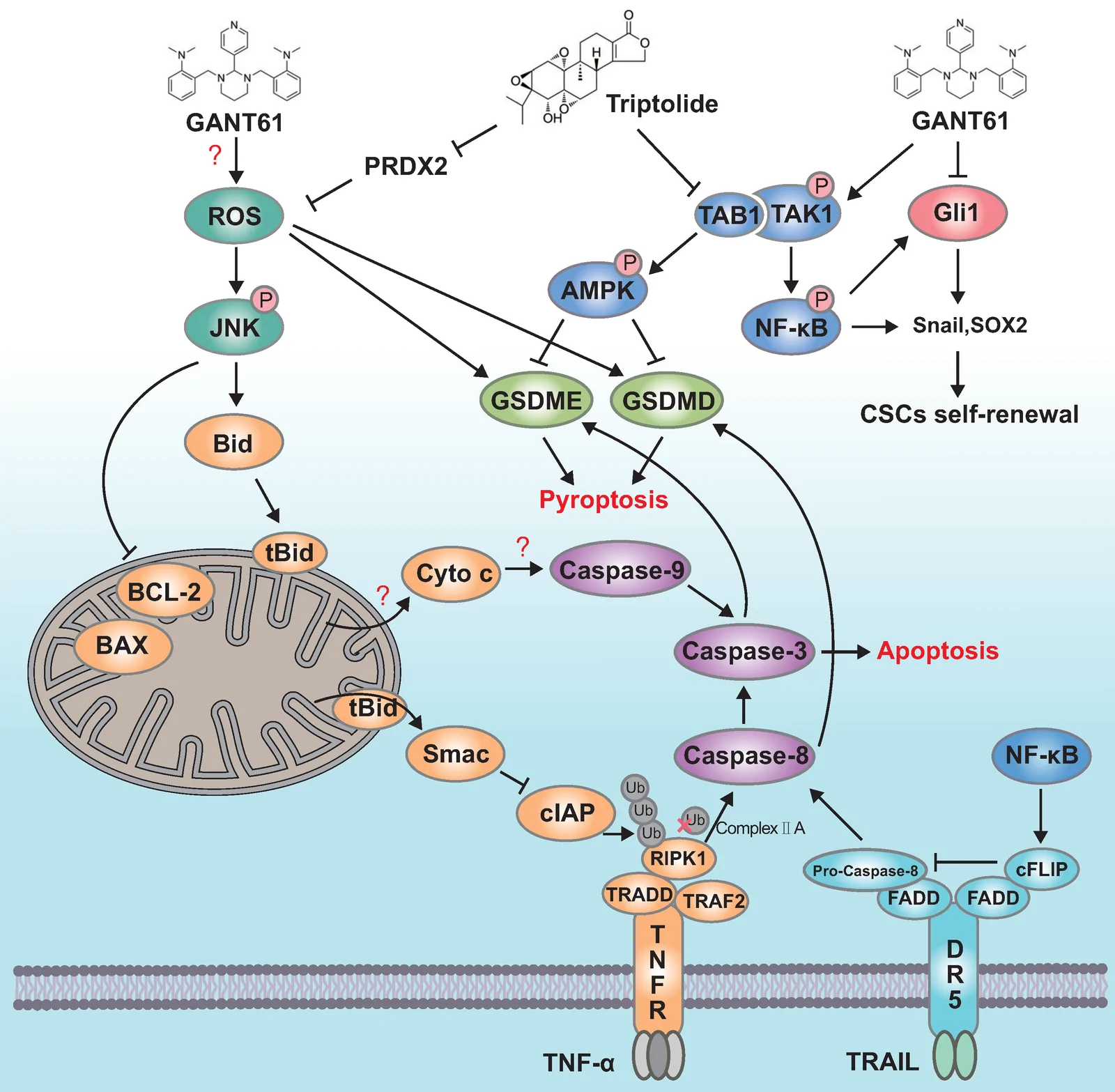
** Schematic diagram of TPL and GANT61-mediated signaling blockade on ATC.** TPL binds TAB1 to inhibit TAK1 activation and binds Peroxiredoxin 2 (PRDX2) to increase intracellular ROS levels. TAK1 activates NF-κB to induce cFLIP expression. Inhibition of TAK1 by TPL leads to decreased cFLIP expression, which sensitizes tumor cells for caspase-8 activation. JNK activation by ROS induces Bid cleavage to produce tBid, which anchors to the mitochondrial membrane to release Smac into the cytoplasm where it inhibits the activity of cIAP, a E3 ubiquitin ligase that ubiquitinates RIPK1. Without ubiquitination, the RIPK1-FADD-caspase-8 complex is dissociated from the TNF receptor. In the absence of cFLIP, caspase-8 is activated to cleave GSDMD and caspase-3. Caspase-3 then cleaves and activates GSDME. GSDMD/E are further activated by ROS-mediated palmitoylation but inhibited by AMPK-mediated phosphorylation. We propose that TPL induces apoptosis and pyroptosis by concomitantly targeting the TAB1-TAK1-NF-κB-cFLIP pathway and the PRDX1-ROS-JNK-tBid-Smac-cIAP pathway. While GANT61 also activates the ROS-JNK-tBid-Smac-cIAP pathway, it poorly induces apoptosis and pyroptosis since it does not inhibit but rather activates the TAB1-TAK1-NF-κB-cFLIP pathway.

## Data Availability

Data is provided within the manuscript or supplementary information files.
