# Supplementary material for: Triptolide in Combination with the Gli1 Inhibitor GANT61 Eradicates Anaplastic Thyroid Cancer by Suppressing Cancer Stem Cell Self-Renewal and Enhancing Apoptosis and Pyroptosis
Source: Int J Biol Sci. 2026 Sep 10;22(14):8008–28. doi: 10.7150/ijbs.131070 (PMC13618228; doi:10.7150/ijbs.131070)

Fig. S1

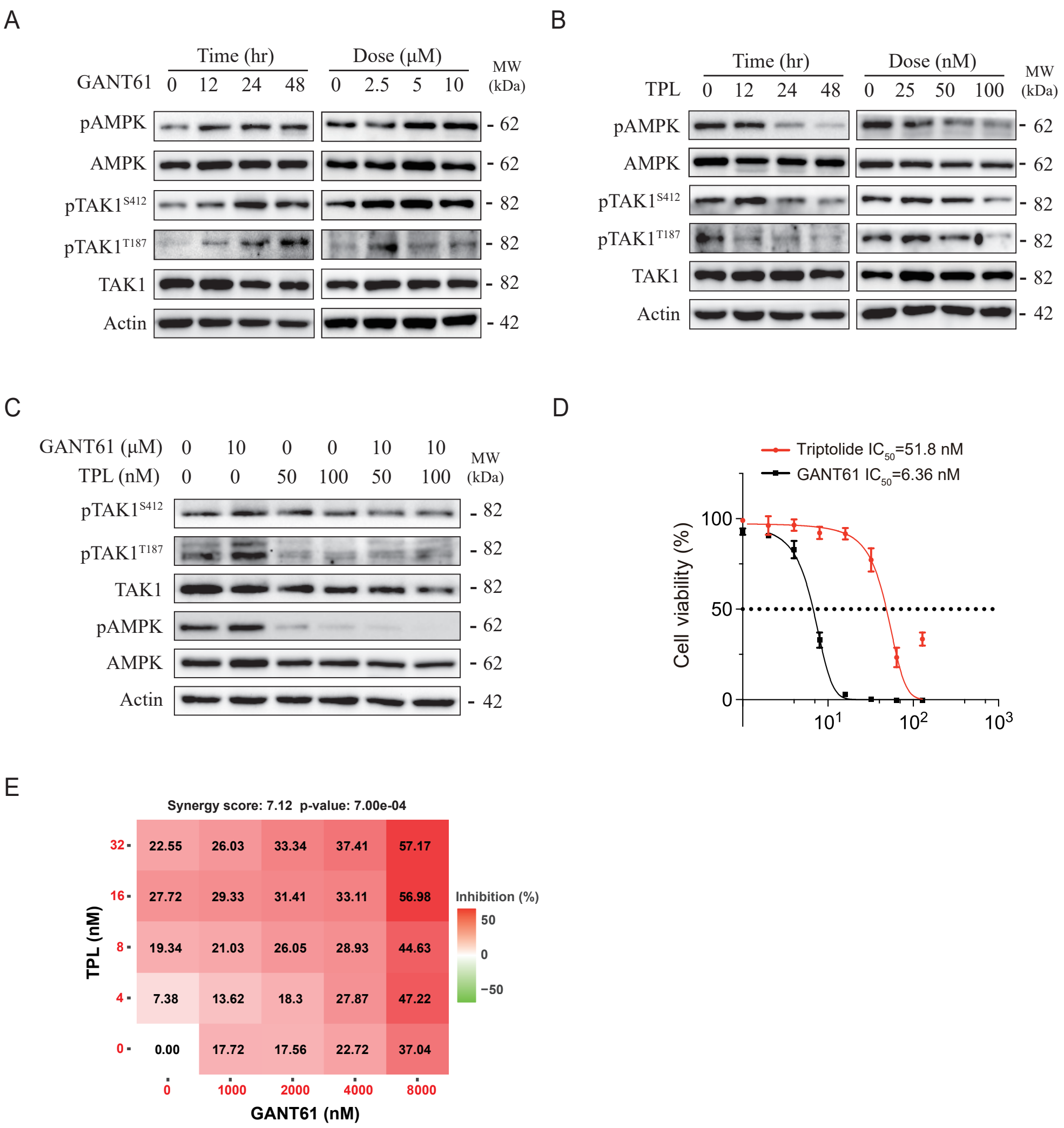

Fig. S1. The effect of TPL and GANT61 on TAK1 phosphorylation and cell proliferation. (**A & B**) WRO82 cells seeded in 12-well plates were treated with GANT61 (10 μM) or TPL (100 nM) for the indicated lengths of time or with the indicated concentrations of GANT61 or TPL for 48 h. (**C**) WRO82 cells were incubated in the absence or presence of GANT61 (10 μM) minus or plus TPL (50 or 100 nM) for 48 h. Cell lysates were prepared and analyzed for phosphorylation of $TAK1^{S412}$, phospho-$TAK1^{T187}$, AMPK, p65 as well as their total corresponding proteins by Western blot. The results represent one of three independent experiments. (**D**) WRO82 cells seeded in a 96-well plate (2000 cells/well) were incubated with the indicated concentrations of TPL or GANT61 for 72 h. Cell proliferation was determined using a CellTiter-Glo kit. The IC_{50} values represent the mean ± standard deviation (SD) of three independent experiments, each in triplicate. (E) WRO82 cells were incubated in the absence or presence of the indicated concentrations of TPL and GANT61 for 48 h and then evaluated for cytotoxicity by LDH release assay.

Fig. S1

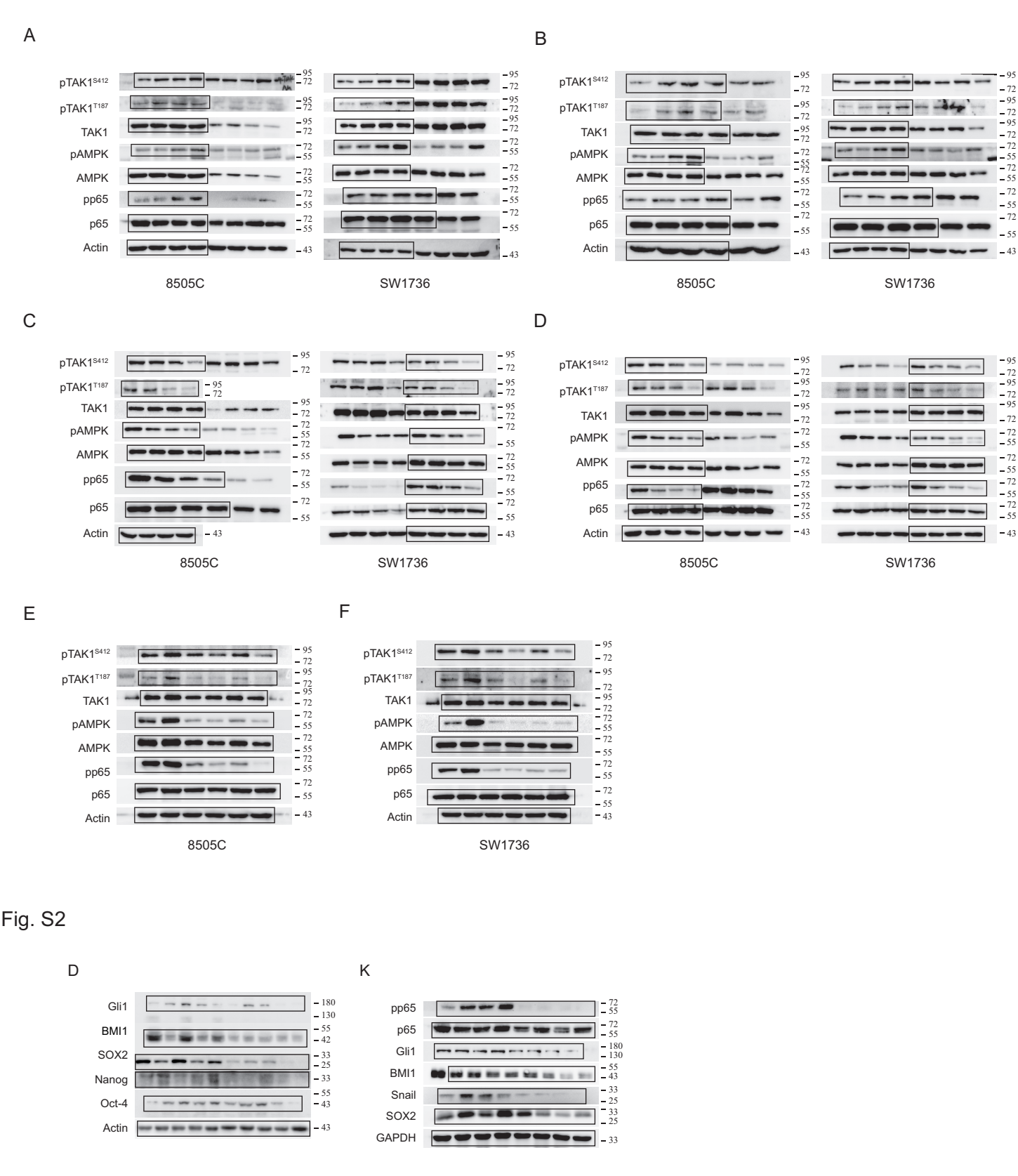

Fig. S2

Fig. S3

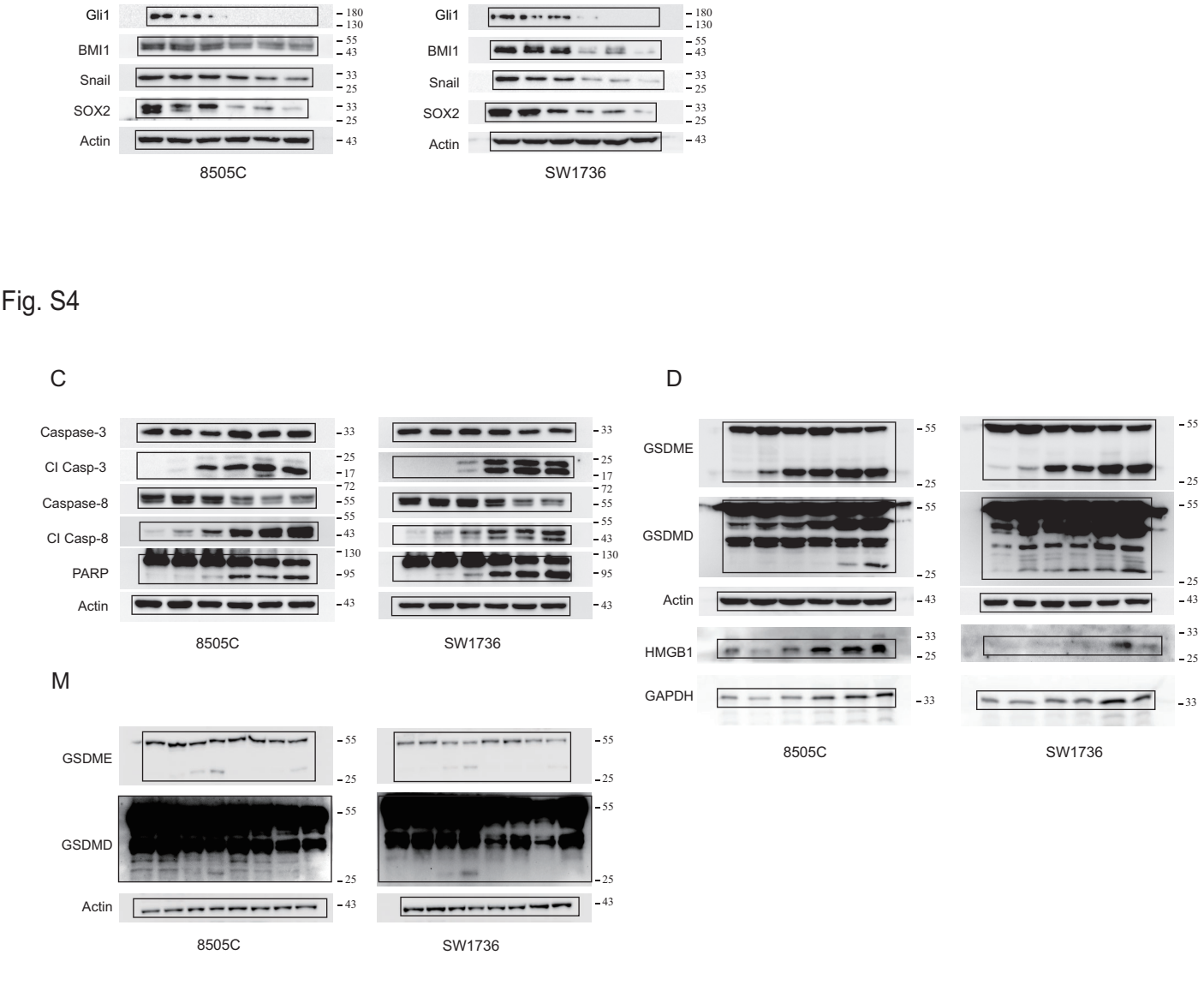

Fig. S4

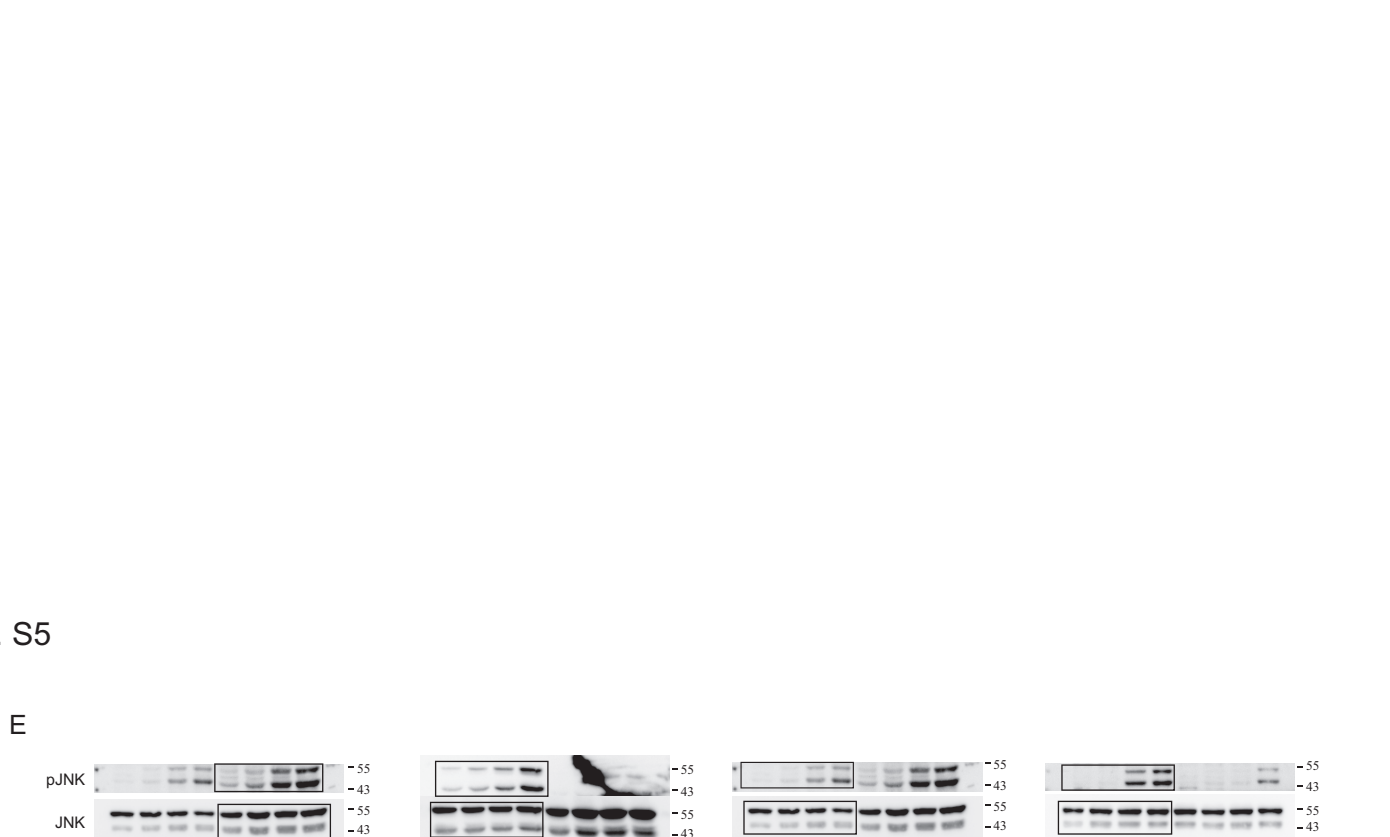

Fig. S5

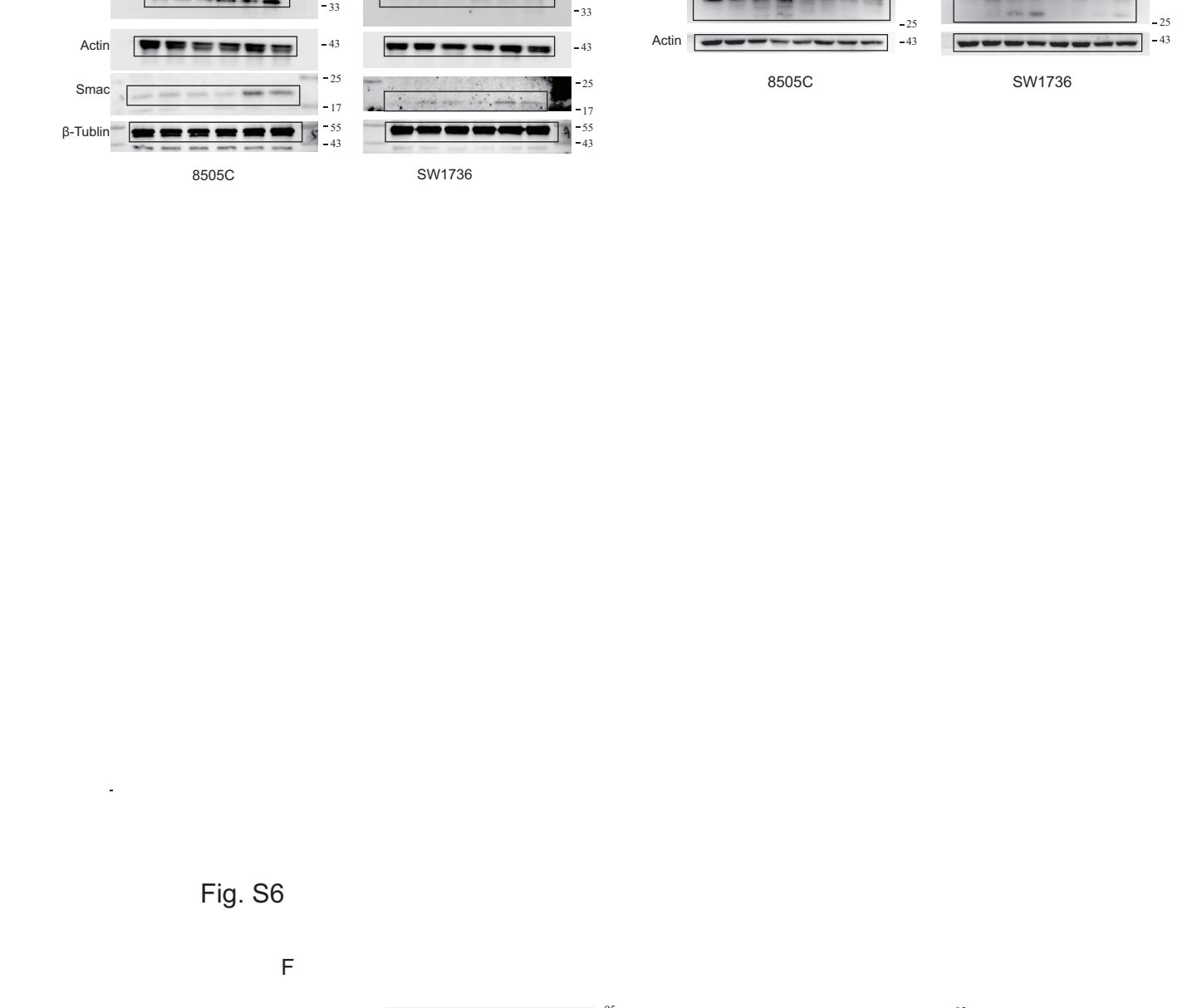

Fig. S6

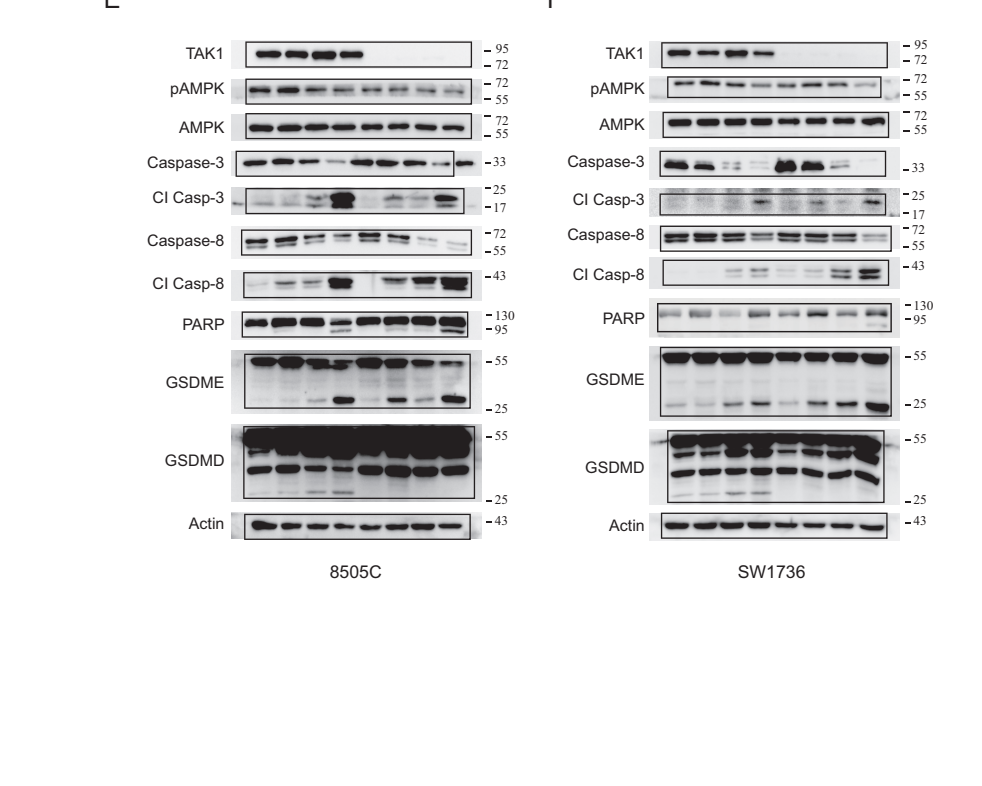

Fig. S7

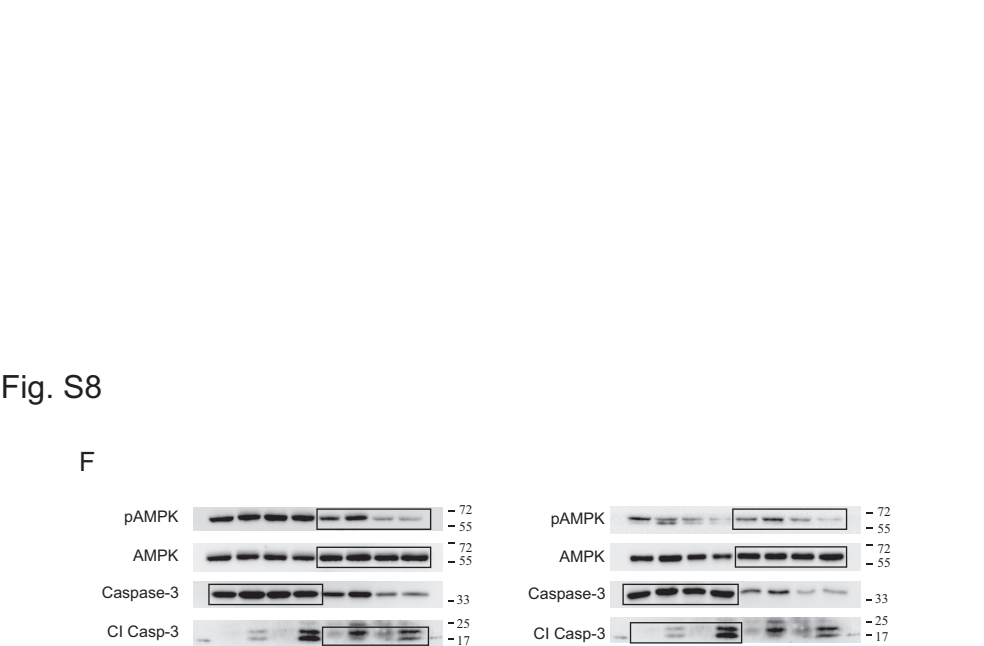

Fig. S8

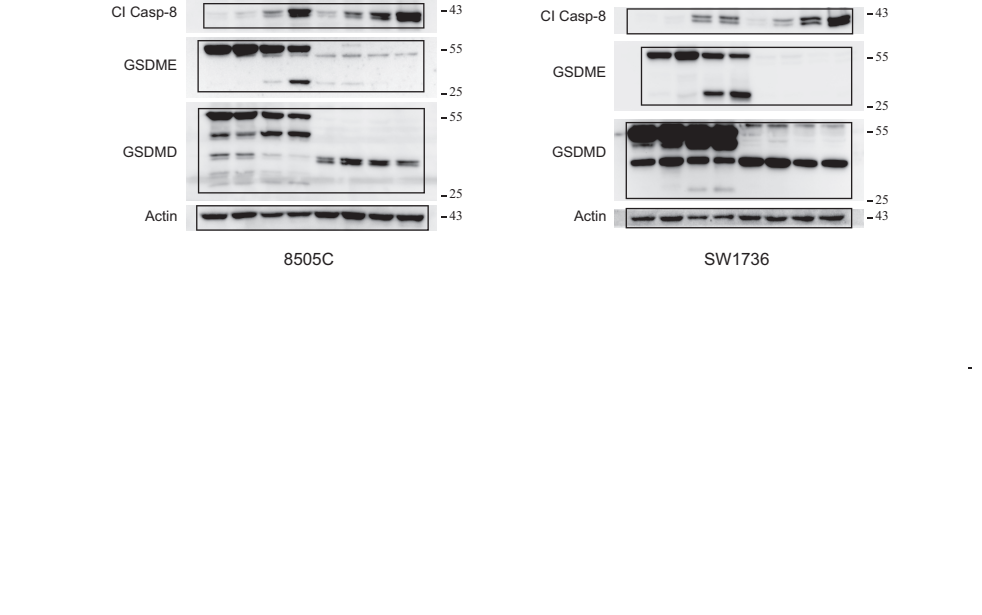

Fig. S9

Fig. S1

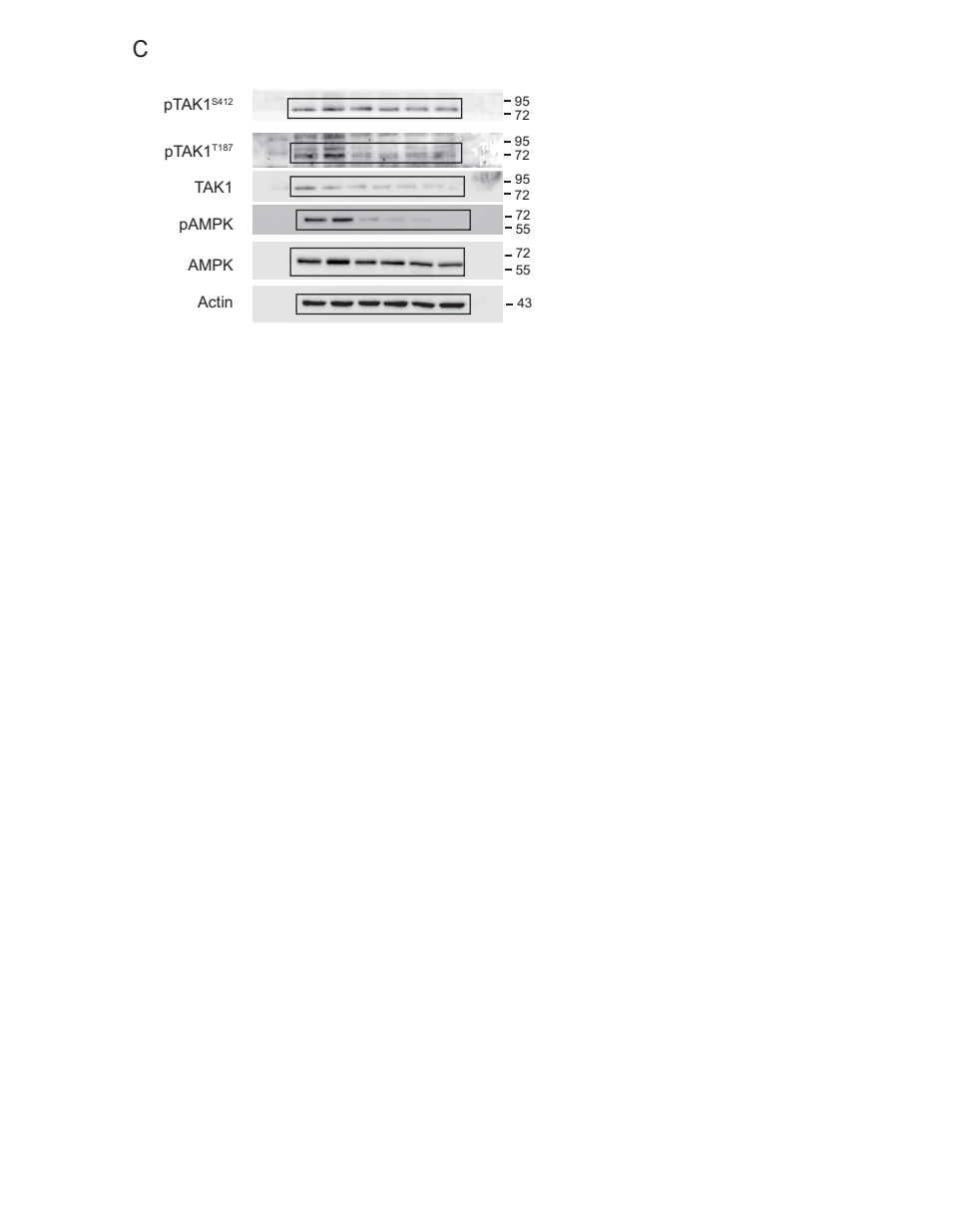

Fig. S4-I

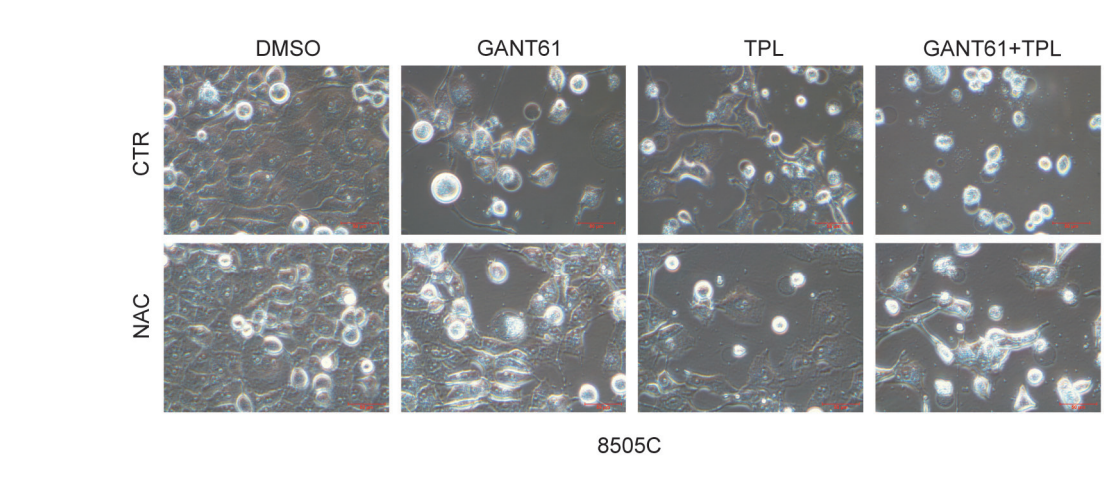

Fig. S5-I

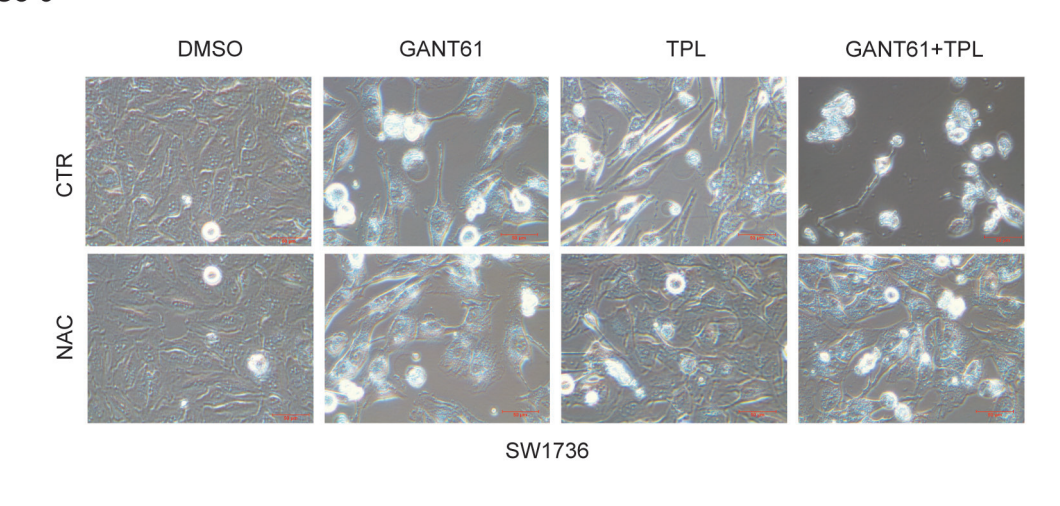

Fig. S5-J

Fig. S6-B & D

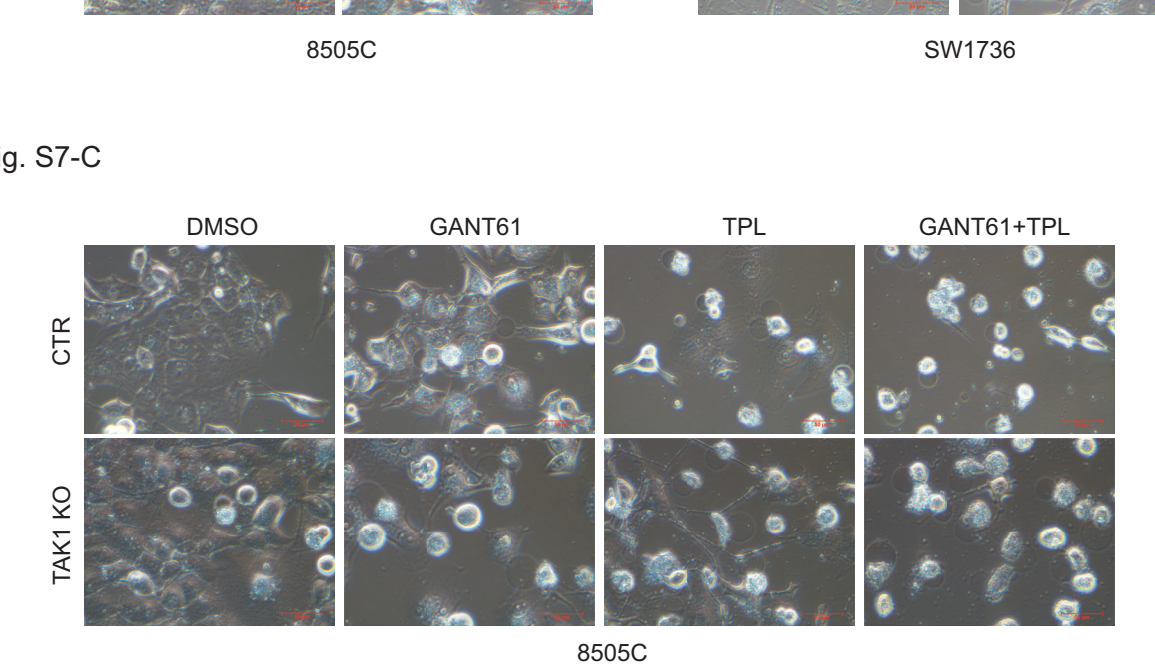

Fig. S7-C

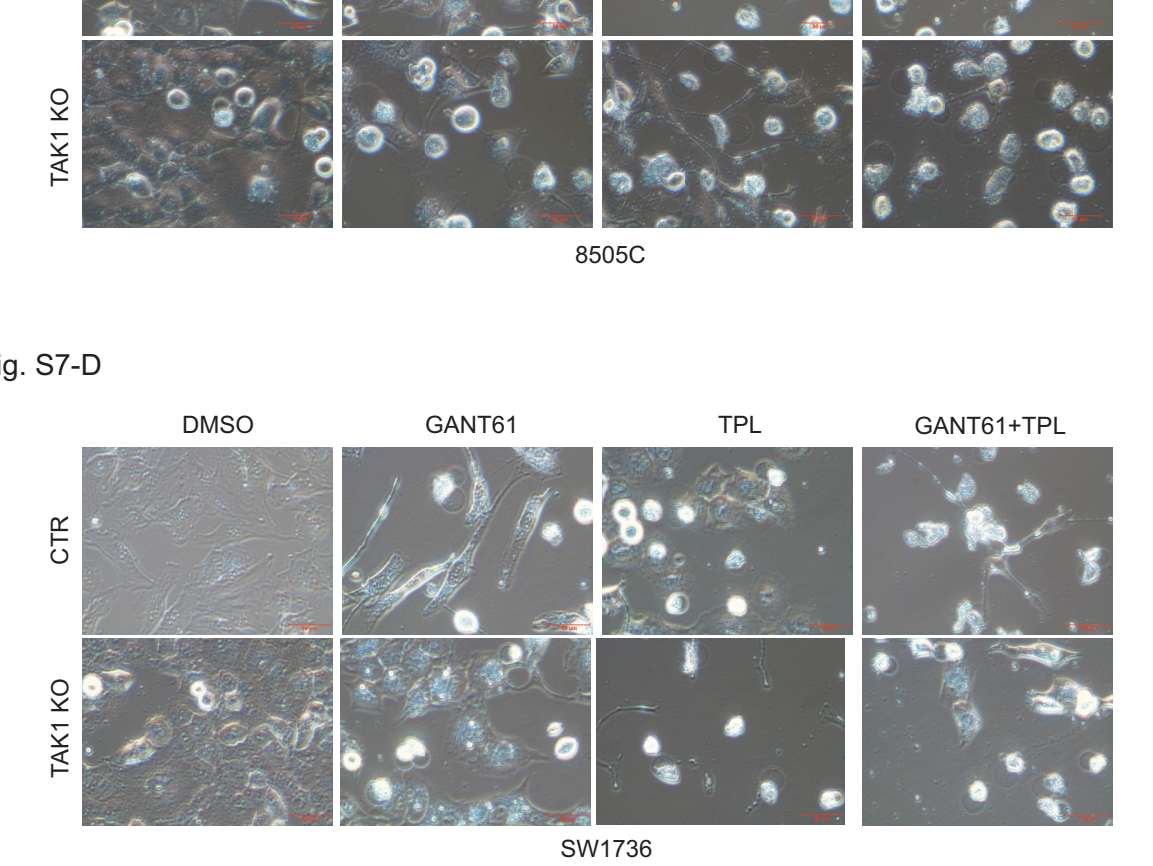

Fig. S7-D

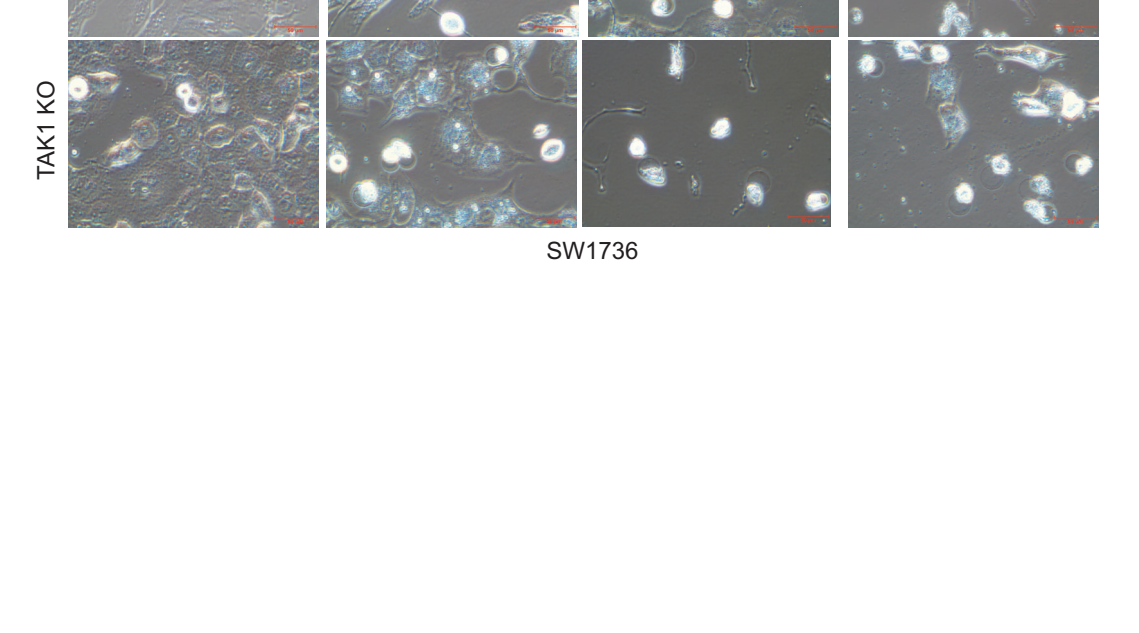

Fig. S8-B & D

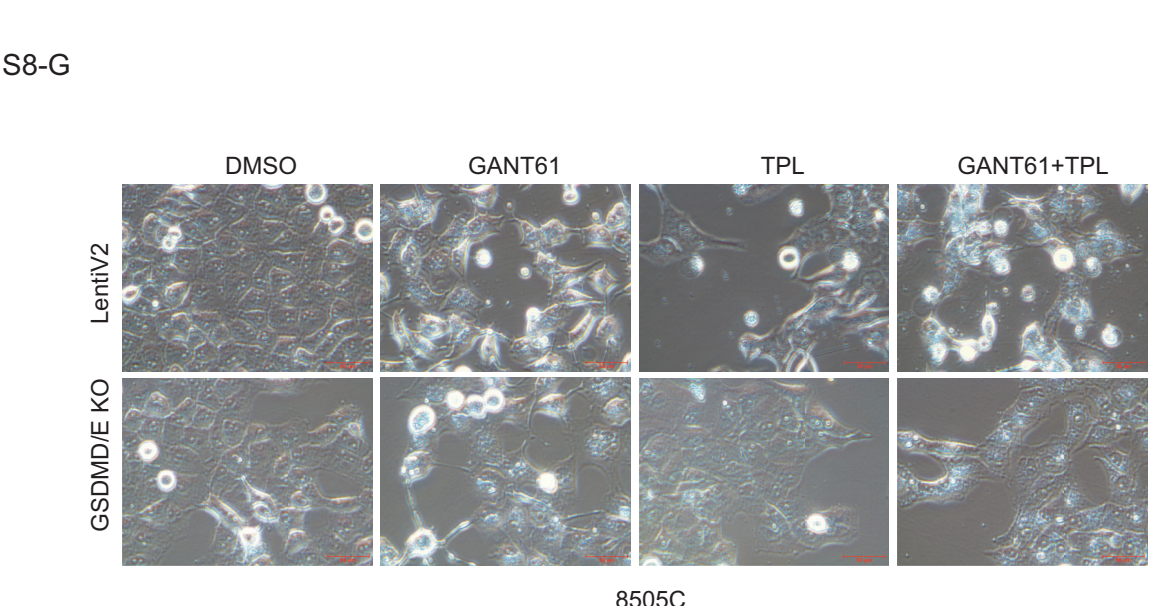

Fig. S8-G

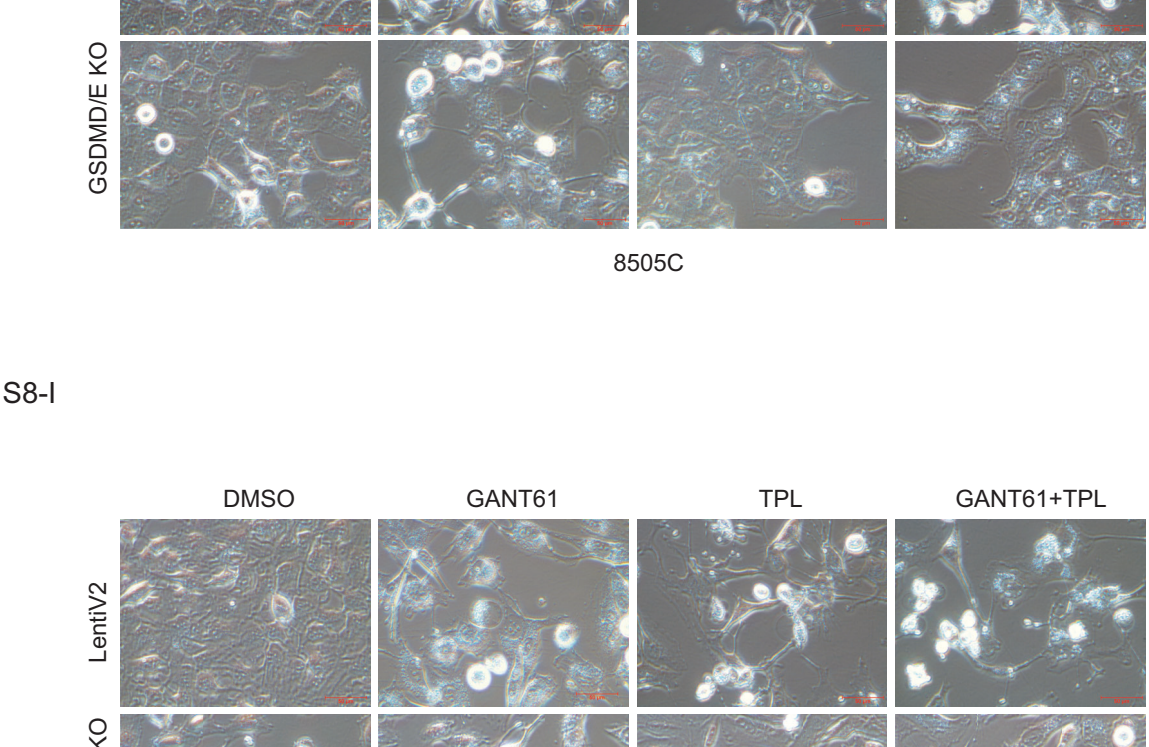

Fig. S8-I

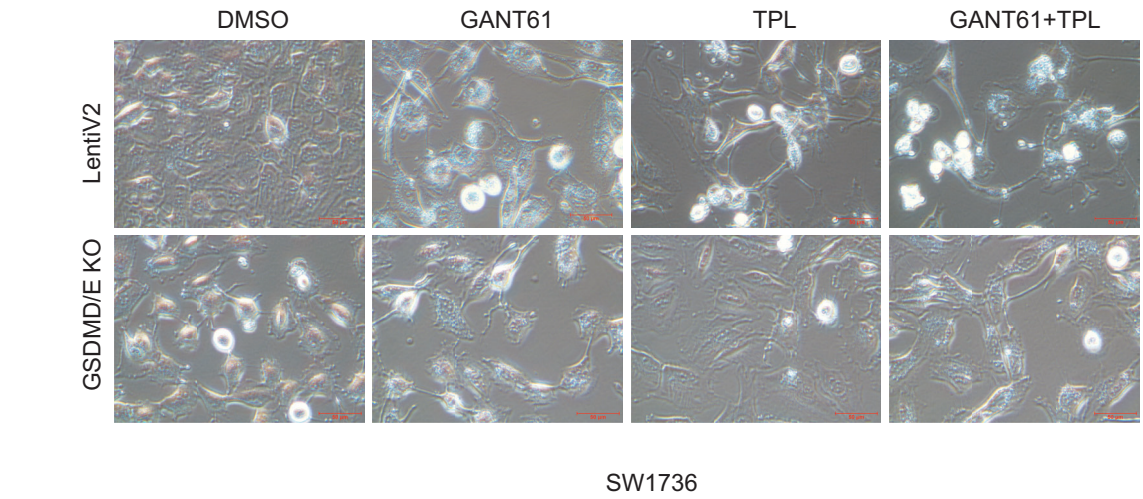

Supplement: Supplementary file 1 [file ijbsv22p8008s1.pdf]
